# Sulfur oxidation and implications for oxygen consumption in Base Mine Lake, the first pilot oil sands pit lake in the Athabasca oil sands region

**DOI:** 10.3389/fmicb.2025.1662147

**Published:** 2025-10-21

**Authors:** James L. S. Arrey, Yunyun Yan, Tara Colenbrander Nelson, Lauren E. Twible, Rui Zhang, Alexandre J. Poulain, Lesley A. Warren

**Affiliations:** ^1^Department of Civil and Mineral Engineering, University of Toronto, Toronto, ON, Canada; ^2^Department of Biology, University of Ottawa, Ottawa, ON, Canada

**Keywords:** sulfur oxidizing bacteria, oil sands tailings reclamation, anoxia, oil sands pit lake, SOB pathways

## Abstract

Base Mine Lake (BML) is the first pilot scale oil sands pit lake in the Athabasca Oil Sands Region (AOSR). Following a whole lake alum addition in September of 2016, a seasonally recurring zone of anoxia developed in the late summer hypolimnion of the BML water cap. The extent to which sulfur cycling exacerbates or mitigates this phenomenon in BML remains unclear. The objective of this 7–year was to characterize the identity and function of the sulfur oxidizing bacteria (SOB) and determine SOB risks to oxygen consumption in BML. The study revealed a persistent community of SOB that collectively encoded the genes involved in the primary sulfur oxidation pathways (Sox, rDSR, and S_4_I). The majority of SOB in BML have been previously identified as heterotrophs, allowing for metabolic flexibility depending on geochemical conditions that varied seasonally. The relative abundance of SOB genera comprising this community shifted as a result of the alum addition and has yet to stabilize over time. Simultaneous consumption of thiosulfate and nitrate was observed in the summer hypolimnion of BML post-alum, consistent with anaerobic sulfur oxidation. Furthermore, the anoxic zone occupied the largest portion of the hypolimnion when anaerobic sulfur oxidation was limited, suggesting it had a mitigating effect on anoxic zone expansion through removal of reduced sulfur species via nitrate driven sulfur oxidation by SOB. Constraining biological impacts to oxygen consumption in this pilot OSPL will be key to managing the growing tailings inventory of the AOSR as another ~23 OSPLs are proposed pending the outcome of BML.

## 1 Introduction

Currently there are over 1.2 billion m^3^ of fluid fine tailings (FFT) that require reclamation in the Athabasca Oil Sands Region (AOSR) (CEC, 2020). FFT, a waste product of oil extraction, consists of oil sands processing water, residual bitumen, and a solids (sand and clay) content ranging from 2 to 30% (Lalonde et al., 2020). One proposed FFT reclamation strategy is water capped tailings technology (WCTT) for which FFT are deposited in mined out pits, and capped with water columns for long-term consolidation, establishing mine-closure landscapes known as oil sands pit lakes (OSPL; CAPP, 2021). The viability of WCTT as an effective reclamation method for oil sands tailings is being examined in the first pilot scale OSPL in the AOSR, Base Mine Lake (BML). Commissioned in 2012, BML has a surface area of ~800 hectares and began with an 8 m water cap covering a 45 m deep layer of FFT which has since consolidated resulting in an average water cap depth of approximately ~12 m (2021).

A metric for OSPL reclamation success is the establishment of habitats that can support native aquatic and terrestrial fauna (CAPP, 2021), for which an oxic zone must persist in the water cap. In early stage development of BML (2015–2016), the oxidation of reduced compounds (e.g., CH_4_, ${\text{NH}}_{4}^{+}$) mobilizing from the FFT into the water cap was identified to impair summer BML water cap dissolved oxygen (DO) levels in the hypolimnion; however this impact was offset by metalimnetic oxygen inputs, maintaining hypolimnetic DO albeit at low concentrations (< 10 μM at the FFT-Water interface [FWI]; Arriaga et al., 2019; Risacher et al., 2018). In September of 2016, a whole lake alum (aluminum sulfate) addition aimed at the removal of suspended solids was done thereby improving water clarity, light penetration, and photosynthesis, consequently increasing surface DO levels. However, a post-alum increase in algal biomass also resulted in greater aerobic heterotrophy, exacerbating oxygen consumption, resulting in ~2 m of anoxia in the hypolimnion during late summer stratification (Jessen et al., 2022). This significant transition point in BML development enabled anaerobic microbial activity, especially sulfide generation via dissimilatory sulfate reduction, within the BML water cap during the late summer anoxic episodes post-alum addition (Jessen et al., 2022), which can rapidly consume oxygen abiotically or through microbial activity. The detection of diverse reduced S species such as sulfide (ΣH_2_S, ≤15 μM), thiosulfate (≤178 μM), and sulfite (≤193 μM) in the BML water column post-alum addition (Yan et al., 2022), indicates possible activity of sulfur oxidizing bacteria (SOB) impairing DO conditions in BML.

Current studies have identified three primary sulfur oxidation pathways used by SOB (1) the sulfur oxidation (Sox) pathway including the complete sox (cSox) and incomplete Sox (iSox) variations, (2) reverse dissimilatory sulfite reductase (rDSR) pathway, and the Kelly-Trudinger (S_4_I) pathways (Whaley-Martin et al., 2023). The cSox pathway is distinguished by the presence of the complete Sox complex including *soxCD* resulting in the complete oxidation of both sulfur atoms in thiosulfate to sulfate (Friedrich et al., 2001). The iSox pathway lacks *soxCD*, which prevents the complete oxidation of thiosulfate through the Sox complex. In organisms with the iSox pathway, the unoxidized sulfane sulfur atom may be transferred to a sulfur globule (S^0^) where persulfides may be removed to cross the cell membrane and participate in other pathways (Frigaard and Dahl, 2008; Meyer et al., 2007). Often paired with the iSox pathway is the rDSR pathway. The rDSR pathway is the reverse reaction that is used by sulfur reducers and typically oxidizes sulfide and elemental sulfur through several genes including *dsrAB*, generating sulfite that can be subsequently oxidized by genes such as *aprAB* and *sat* (Kappler and Dahl, 2001; Loy et al., 2009; Meyer and Kuever, 2007). The S_4_I pathway is known for producing and consuming tetrathionate as an intermediate (Hutt et al., 2017). Some common genes involved are *tsdA*, and *doxDA* (*tqo*) both of which oxidize thiosulfate to tetrathionate (Brito et al., 2015; Kanao, 2024). Less common is *tetH*, which disproportionates tetrathionate into sulfate, thiosulfate, and elemental sulfur (Meulenberg et al., 1993; Watanabe et al., 2019). Further, SOB that possess only partial or single genes associated with sulfur oxidation have also been noted to oxidize sulfur. For example, SOB possessing *soxCD* but lacking many of the other critical genes associated with the *sox* pathway including *soxB*, have been observed to oxidize elemental sulfur to thiosulfate and sulfate (Lahme et al., 2020).

The BML water cap SOB community structure and sulfur oxidation pathways, important for water cap O_2_ persistence, have yet to be elucidated. Thus, the objectives of this study were to investigate the composition, metabolic potential, and physico-geochemical driving factors of the endemic SOB community in BML and how these were related to physicochemical and geochemical characteristics over annual, seasonal, and spatial scales across a 7–year time frame (2015–2021) that bracketed the whole lake alum addition (September 2016). Clarifying the impact of SOB on the water cap DO concentrations of BML will be key to determining the long-term success of the WCTT for FFT reclamation.

## 2 Methods

### 2.1 Site description

**Figure 1:**
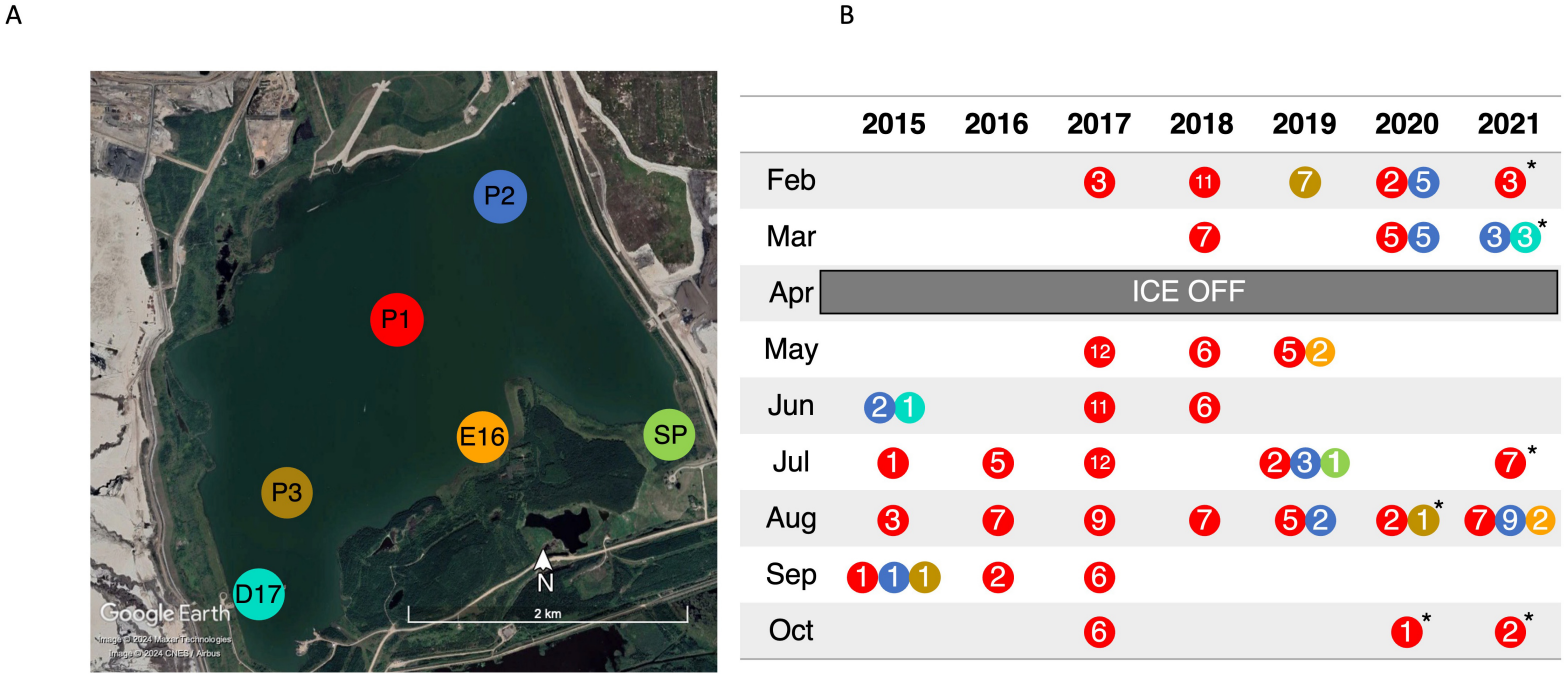
**(A)** Aerial view of Base Mine Lake with sampling points marked. Image obtained from Google Earth Pro Version (2021). **(B)** Summary of 16S rRNA samples collected over the course of the study. Colors represent the sampling location marked on panel a, while the number indicates the number of depths collected that month. ^*^ indicates samples were shipped to the University of Toronto before subsampling and analysis.

BML is located on the Mildred Lake mine in the AOSR (57.011553, −111.622203), and in 2021 consisted of approximately 40 m of tailings beneath a 12 m water cap. Tailings deposition halted in 2012, and oil sands processing water (OSPW) as well as fresh water from a nearby reservoir (Beaver Creek Reservoir) was added to create a water cap 8 m deep. Due to FFT consolidation the water cap maximum depth has increased to 13 m in 2021. The lake is dimictic with summer and winter thermal stratification. Ice usually begins to form on the surface in November and lasts until April. After spring turnover (April–June), the lake thermally stratifies with peak stratification usually occurring in mid-late August, and fall turnover, typically occurring in late August to early September (Tedford et al., 2019). There are continuous inputs of water from Beaver Creek Reservoir to account for evaporation and to maintain a constant elevation 308 m above sea level (Syncrude Canada Ltd, 2021). Three sampling platforms (P1, P2, and P3) exist on BML (Figure 1) from which samples are taken.

### 2.2 Sample collection and physicochemical characterization

Between 2015–2021, 32 sampling campaigns were carried out during which 200 depth dependent samples were collected for geochemical and 16S rRNA analysis (Figure 1). During most sampling campaigns, samples were collected from several depths from the surface to the FFT-water interface (FWI) at P1 (156 samples) with the addition of other sites including P2 (30 samples), P3 (9 samples), D17 (4 samples), E16 (4 samples), and SP (1 sample) in 2015 and 2019–2021 (Figure 1B).

Physicochemical characterization and sample collection followed established protocols in Risacher et al. (2018) and Whaley-Martin et al. (2020). Dissolved oxygen (DO), temperature (°C), and pH were determined using a YSI ProDSS multiprobe approximately every 0.5 m from the water cap surface to the FWI. Sampling depths representing varied physicochemical conditions were then collected using a Van Dorn water sampler (WaterMark, Forestry Suppliers Inc.; Wildco Beta Plus, Wildlife Supply Company ^®^). The DO probe used was accurate to ± 0.1 mg/L and calibrated to both 100% and 0% DO saturation prior to use. Water samples for [Total S], [${\text{SO}}_{4}^{\text{2}-}$], [${\text{SO}}_{3}^{\text{2}-}$], [S_2_${\text{O}}_{3}^{\text{2}-}$], [ΣH_2_S], [${\text{NO}}_{3}^{-}$], [${\text{NO}}_{2}^{-}$], and [Total organic carbon] were taken directly on the boat. Water samples for 16S were transferred to polyethylene bags that were previously rinsed with 70% ethanol before being triple rinsed with sample water before filling. The bag was immediately sealed such that there was no headspace and then placed in a clean 20 L container for transport back to the onsite laboratory. Due to COVID restrictions, from August 2020 to July of 2021, bulk water samples were collected in bags as described above and shipped to the University of Toronto for subsampling.

### 2.3 Geochemical analyses

The sampling and measurement of [ΣH_2_S], [${\text{SO}}_{4}^{\text{2}-}$], [S_2_${\text{O}}_{3}^{\text{2}-}$], [${\text{SO}}_{3}^{\text{2}-}$], [Total S] followed detailed protocols described in Whaley-Martin et al. (2020) and Yan et al. (2022). Briefly, [ΣH_2_S] was measured immediately according to the USEPA Standard Method: 4500-S2-D (Methylene Blue Method, Hach Method 8131) using a Hach DR1900 spectrophotometer. The detection limit (DL) was determined to be 40 ug/L and 10 ug/L was subtracted from every sample to account for the water color of BML following (Yan et al., 2022). Samples for anions including ${\text{SO}}_{4}^{\text{2}-}$, ${\text{NO}}_{3}^{-}$, and ${\text{NO}}_{2}^{-}$ were collected, filtered, and preserved according to protocols outlined in Arriaga et al. (2019) and Yan et al. (2022). Briefly the samples were centrifuged and 0.45 μM filtered using Pall Acrodisc^®^ PES syringe filters (2015–2016; Arriaga et al 2019) or 0.2 μM filtered (2017–2021) using aPES filters (Thermo scientific™ Nalgene™ Rapid-Flow™, 2017–2019; Pall Acrodisc^®^, 2019–2021; Yan et al., 2022) before being stored at 4 °C until analysis. [${\text{SO}}_{4}^{\text{2}-}$] was measured using a Hach DR1900 spectrophotometer following USEPA method 375.4 (2015–2019) or using an ion chromatography (IC) system (Dionex ICS-6000 Capillary HPIC™, Thermo Scientific™) following USEPA method 300.0 and 300.1 (2019–2021) according to Yan et al. (2022). Similarly, from 2015 to 2019 [${\text{NO}}_{3}^{-}$] and [${\text{NO}}_{2}^{-}$] were analyzed using a Hach DR1900 spectrophotometer following Hach method 8171 (${\text{NO}}_{3}^{-}$) and USEPA method 354.1 (${\text{NO}}_{2}^{-}$). From 2019 to 2021, [${\text{NO}}_{3}^{-}$] and [${\text{NO}}_{2}^{-}$] were analyzed using Dionex ICS-6000 Capillary HPIC™ (Thermo Scientific™) following USEPA method 300 and 300.1. As described in Yan et al. (2022), all anions were chromatographically separated by a Dionex IonPac™ AS18-Fast anion exchange column (7.5 μm, 4 x 150 mm, Thermo Scientific™) and quantified based on calibration curves derived from commercial IC stock standard (Inorganic Ventures, USA).

For S_2_${\text{O}}_{3}^{\text{2}-}$ and ${\text{SO}}_{3}^{\text{2}-}$ analysis, unfiltered water samples were monobromobimane derivatized on the boat according to protocols described by Rethmeier et al. (1997) and Whaley-Martin et al. (2019), before being stored at −20 °C until analysis. The [S_2_${\text{O}}_{3}^{\text{2}-}$] and [${\text{SO}}_{3}^{\text{2}-}$] were determined using a Prominence HPLC-Florescence system (Shimadzu, Japan) following protocols in Yan et al. (2022). The detection limit for both S_2_${\text{O}}_{3}^{\text{2}-}$ and ${\text{SO}}_{3}^{\text{2}-}$ was 5 μM (Yan et al., 2022). Dissolved total sulfur samples (0.45 μm filtered) were preserved in 0.2% HNO_3_ and stored at 4 °C until analysis. As described in Yan et al. (2022) samples were analyzed using inductively coupled plasma atomic emission spectroscopy (ICP-AES, Vairan730 ES, Varian Inc.; 2015–2019) or inductively coupled plasma optical emission spectroscopy (ICP-OES, iCAP™ 7000 Series, Thermo Scientific™; 2019-2021). The detection limit was found to be 0.1 mg/L. Total S concentrations in conjunction with sulfate concentrations, were used to determine reactive sulfur (Sreact; Whaley-Martin et al., 2020), which was calculated by subtracting [${\text{SO}}_{4}^{\text{2}-}$] from [Total S]. This gives a measure of all sulfur atoms regardless of speciation that are oxidizable. Sreact values were calculated for 156 samples from 2015–2021. Samples taken for total organic carbon (TOC) and dissolved organic carbon (DOC) were collected in acid washed and pre-combusted (450 °C, 8 h) glass vials and stored at −20 °C until analysis at the University of Toronto as described in Whaley-Martin et al. (2023). Samples for dissolved organic carbon (DOC) were syringe filtered through a Pall Acrodisc^®^ 25 mm 0.45 μm Supor^®^ membrane filter using a polypropylene syringe and analysis for total carbon and inorganic carbon was carried out on a Shimadzu TOC-L. TOC and DOC values were determined by subtracting the concentration of inorganic carbon from the concentration of total carbon for unfiltered and 0.45 μm filtered samples respectively.

### 2.4 DNA extraction

Approximately 0.5–3 L of sample water was filtered using 0.2 μm or 0.1 μm aPES filters (Thermo Scientific™ Nalgene™ Rapid-Flow™ sterile vacuum filter units) until the filter was clogged. The filters were then excised and stored at 20 °C or −80 °C until DNA extraction using a QIAGEN DNeasy PowerWater Kit according to their protocols. The extracted DNA was then sent to the McMaster DNA Sequencing Facility (Hamilton, Ontario, Canada) and Genome Quebec (Montreal, Quebec, Canada) for further analysis.

### 2.5 Amplicon analysis of the 16S rRNA gene

Aliquots of purified DNA were used to amplify the V4 region of the 16S rRNA gene by PCR using Illumina adapted primers (Bartram et al., 2011). Primers 515 F (Parada) and 806 R (Apprill) were used to target both bacterial and archaeal DNA. PCR was performed using 50 ng of the template and the PCR mix containing 1U of recombinant Taq DNA Polymerase (Invitrogen™), 1x buffer, 1.5 mmol/L MgCl2, 0.4 mg/mL BSA, 0.2 mmol/L dNTPs, and 5 pM of each primer. The reaction was carried out at 98 °C for 5 min, 35 cycles (98 °C) for 30 s, then 30 s at 50 °C and 30 s 72 °C, with a final extension of 72 °C for 10 min. PCR products were checked by electrophoresis. All amplicons were normalized using the SequalPrep normalization kit (ThermoFisher#A1051001) and sequenced using the Illumina MiSeq platform. Raw sequences were filtered and trimmed with a minimum quality score of 30 and a minimum read length of 100 bp using Cutadapt (Martin, 2011). DNA sequence reads were filtered and trimmed based on the quality of the reads for each Illumina run separately, error rates were learned, and sequence variants were determined by DADA2 version 1.6.0 (Callahan et al., 2016). Bimeras were removed and the SILVA taxanomic database version 138.1 (Quast et al., 2013) was used to assign taxonomy using on 16S rRNA sequences.

### 2.6 Metagenomic analysis

Extracted DNA samples were sent to the McMaster University Farncombe Genome Facility (Hamilton, Canada) and Genome Quebec (Montreal, Canada) for metagenomic sequencing. Sample extracts were dried and resuspended in 25 μL of water before construction of libraries and sequencing by Illumina HiSeq 1,500 with paired-end 150 bp sequencing following protocols in Whaley-Martin et al. (2023). Bioinformatic analyses was completed at the Center for Advanced Research in Environmental Genomics, University of Ottawa (Ottawa, Canada) according to the protocols outlined in Zhang R. et al. (2023) and Yan et al. (2025). Briefly raw paired-end reads were filtered using fastp (v 0.23.1; Chen, 2023) before being assessed for quality in FastQC (v 0.11.0; Andrews, 2010). DNA reads from each sample were then assembled using MetaSPAdes (v 3.15.5, default k-mer parameters; Nurk et al., 2017). MEGAHIT (v 1.2.9; Li et al., 2015) was used to carry out co-assembly of metagenomic short reads. Co-assembled contigs were trimmed using Anvi'o (v7.1; Eren et al., 2021) with any less than < 2000 kb discarded. BAM files were created by aligning metagenomic short reads to the co-assemblies via BWA-MEM (v 0.7.17; Li, 2013) and SAMtools (v 1.17; Danecek et al., 2021). The binning of raw BAM files was done using the following tools: MetaBAT 2 (v 2.15; Kang et al., 2019), MaxBin 2 (v 2.2.7; Wu et al., 2014), CONCOCT (v 1.10; Alneberg et al., 2014) and VAMB (v 4.1.1; Nissen et al., 2021). Bins were evaluated for quality by CheckM2 (v 1.0.2; Chklovski et al., 2023) with the highest quality bins from each co-assembly chosen by DAS-Tool (v 1.1.6; Sieber et al., 2018). Bins that were selected by DAS-Tool and passed the CheckM2 filtering were then used as Metagenome Assembled Genomes (MAGs). The Genome taxonomy database (GTDB release 214; Parks et al., 2021) was used to assign MAG taxonomy via GTDB-Tk (v 2.3.0; Chaumeil et al., 2022). The protein coding genes from assemblies were determined using Prodigal (v 2.6.3; Hyatt et al., 2010). For annotation of gene function, representatives of the functional guilds within contigs were retrieved using Hidden Markov Models (HMMs) from various databases using HMMER (v 3.3.1; http://hmmer.org/)

### 2.7 Statistical analyses

Welch's *t* tests and redundancy analyses (RDA) were carried out using R version 3.6.0 with the Vegan package version 2.6–4 being used for the latter. The significance level used for the Welch's *t* tests was 0.05. Values that were below detection limit were treated as zero for all statistical analyses.

### 2.8 Sulfur oxidizing bacterial enrichments

Sample water for enrichments were placed in sterile 90–150 mL containers with no headspace and stored at 4 °C until enrichments were started. Enrichments were carried out using neutrophilic sulfur oxidizing media (NSOM). The NSOM consisted of 90 mL of 1.1% (w/v) K_2_HPO_4_, 90 mL of 0.44% (w/v) NH_4_Cl, 90 mL of 0.11% (w/v) MgSO_4_, 720 mL of tap water, and 2.2 mL of a trace metal solution (~73 g/L EDTA, 13 g/L NaOH, 7.4 g/L ZnSO_4_ 7H_2_O, 7.4 g/L CaCl_2_, 3.7 g/L MnCl_2_ 6H_2_O, 0.7 g/L CoCl_2_ 6H_2_O, 0.7 g/L ammonium molybdate, 7.4 g/L FeSO_4_ 7H_2_O, 0.3 g/L CuSO_4_ 5H_2_O). Added to the NSOM was sodium thiosulfate and potassium tetrathionate to reach a final concentration of 31.6 mM thiosulfate and 16.5 mM tetrathionate in the media. Once the media was complete, it was filter sterilized using Thermo ScientificTM NalgeneTM Rapid-FlowTM sterile single use vacuum filter units either 0.2 μm or 0.1 μm aPES filter membranes, before being stored at 4 °C until use. Elemental sulfur was also added to the enrichments however, due to its low solubility, the elemental sulfur was weighed directly into the previously acid washed and autoclaved Erlenmeyer flasks before being autoclaved 3 times at ~110 °C, for a minimum of 30 min each, to sterilize it. Elemental sulfur was added to the flasks to reach a concentration of 62.4 mM in the enrichment, however due to low solubility the concentration of dissolved elemental sulfur was lower. Sample water from BML and NSOM were added to enrichment flasks in a 1:1 ratio to create the 1° enrichment. Enrichments were then stored in the dark at room temperature. The pH of the enrichment was regularly measured using a sterilized bench top pH probe inside of a biological safety cabinet. Once the pH of the 1° enrichment decreased to less than pH 5, a new enrichment (2° enrichment) was made by adding fresh NSOM and the previous enrichment in a ratio of 2:1, to a new Erlenmeyer flask that had previously been prepared with elemental sulfur. The new enrichment was then adjusted to pH 7 ± 0.05, using optima HCl and NaOH. The pH of the 2° enrichment was then monitored in the same way and once the pH dropped below 5, the above process was repeated to create a 3° enrichment. Once the 3° enrichment dropped below pH 5 the enrichment was ended, and the communities preserved at −80 °C. At the end of each 3° enrichment samples were taken for 16S rRNA analysis according to the same protocols as used for field samples except a smaller volume (12–13 mL) was filtered.

## 3 Results and discussion

### 3.1 Identity and function of the SOB community

**Figure 2:**
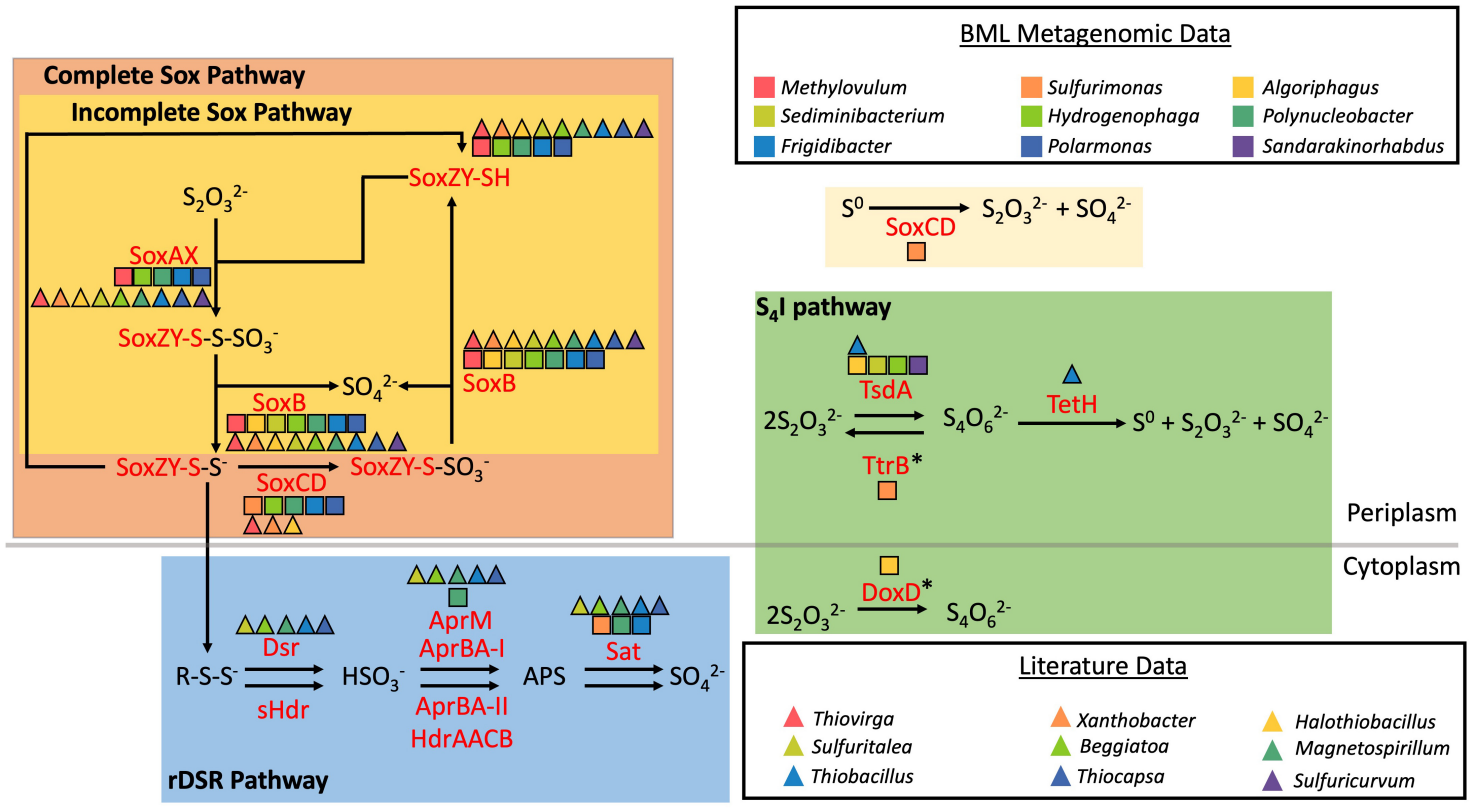
Sulfur oxidation genes possessed by SOB in BML based on metagenomic and literature data (Adapted from Whaley-Martin et al., 2023). *denotes genes shown are part of a complex and not solely responsible for the associated reaction.

16S rRNA relative abundance data were analyzed for 200 BML samples collected over a 7–year period, from 2015 to 2021. A total of 14,712,302 high quality reads from the V4 region of the 16S rRNA gene were acquired from the 200 samples. Each sample averaged 73,562 reads with a minimum of 132 reads and a maximum of 407,830 reads. These were analyzed as amplicon sequence variants (ASVs) rather than operational taxonomic units (OTUs), to increase precision and comparability with other studies (Callahan et al., 2017, 2019; Caruso et al., 2019). Using co-assembled metagenome assembled genome (MAG) data from 45 P1 samples spanning multiple depths to seasons from 2015 to 2020, 17 MAGs, belonging to 9 genera, were investigated for their sulfur oxidation potential. Nine additional SOB genera without corresponding MAG data were metabolically classified based on existing literature (Figure 2; Beller et al., 2006; Geelhoed et al., 2010; Handley et al., 2014; Li et al., 2019; Meyer et al., 2007; Mußmann et al., 2007; Petushkova et al., 2024; Rudenko et al., 2020; Veith et al., 2012; Watanabe et al., 2014; Whaley-Martin et al., 2023). Combined 16S rRNA data and MAG results revealed the SOB community of BML was collectively capable of multiple sulfur oxidation pathways, including the cSox, iSox, rDSR and S4I pathways (Figure 2). MAGs reconstructed for *Sulfurimonas* spp. did not possess a complete set of genes for any one of the above pathways, instead containing *soxCD* exclusively, and thus potentially only capable of oxidizing elemental sulfur to thiosulfate and sulfate (Figure 2). Research on published genomes has found this to be a common occurrence within the genus *Sulfurimonas* (Lahme et al., 2020).

The SOB genera found to occur in BML have been observed in oil contexts in previous studies as well as freshwater, marine, soil/sediment, wastewater, and metal mining environments highlighting their broad ecological relevance and habitat range (Alegado et al., 2013; Arce-Rodríguez et al., 2019; Baker and Banfield, 2003; Biderre-Petit et al., 2011; Golby et al., 2012; Grote et al., 2007; Haosagul et al., 2020; Hubert et al., 2012; Jiao et al., 2018; Jin et al., 2017; Kojima et al., 2014; Lee et al., 2012; Liu et al., 2009; Luo et al., 2018; Matsunaga et al., 1991; Mcilroy et al., 2016; Nedashkovskaya et al., 2004; Postec et al., 2015; Qu and Yuan, 2008; Revathy et al., 2016; Rochman, 2016; Salam et al., 2023; Schleifer et al., 1991; Sethuraman et al., 2022; Stasik et al., 2021; Van den Ende and Van Gemerden, 1993; Van Trappen et al., 2004; Whaley-Martin et al., 2023; Young et al., 2009). While individually these SOB are not exclusive to BML, to the best of our knowledge no other study has reported the occurrence of all these SOB in one context, suggesting BML has a unique and complex SOB community.

Many SOB have demonstrated heterotrophic growth and according to existing literature, instances of non-sulfur energy metabolisms, most commonly heterotrophic, have been reported for at least 16 of the 18 SOB genera identified in BML waters (Asao et al., 2007; Fan et al., 2023; Geelhoed et al., 2010; Güde et al., 1981; Hahn et al., 2010; Han et al., 2017; Han and Perner, 2015; Harrison et al., 1980; Hubert et al., 2012; Jeon et al., 2004; Kodama and Watanabe, 2004; Kojima and Fukui, 2011; Lefèvre et al., 2012; Li and Zhou, 2015; Meijer et al., 1990; Oshkin et al., 2016; Petushkova et al., 2024; Prescott et al., 2002; Sethuraman et al., 2022; Zeng et al., 2013). Possession of both heterotrophic and autotrophic metabolisms, often referred to as mixotrophy, has been previously observed in bacteria, including SOB (Mubarok et al., 2017; Sun et al., 2019). Previous research has identified mixotrophs abundantly grow in environments such as groundwater and boreal lakes, due to their ability to satisfy carbon requirements using both inorganic and organic carbon which gives them increased resistance to changes in labile organic carbon (Heinze et al., 2023; Taubert et al., 2021). Therefore, while there is the potential for these genera to be heterotrophic, the presence of sulfur metabolic capabilities and especially the presence of the entire genetic machinery required to carry out all three primary sulfur oxidation pathways across these SOB (Figure 2) suggests there may be dynamic biogeochemical windows where sulfur oxidation is occurring in BML.

### 3.2 Trends in SOB abundance through time and depth

**Figure 3:**
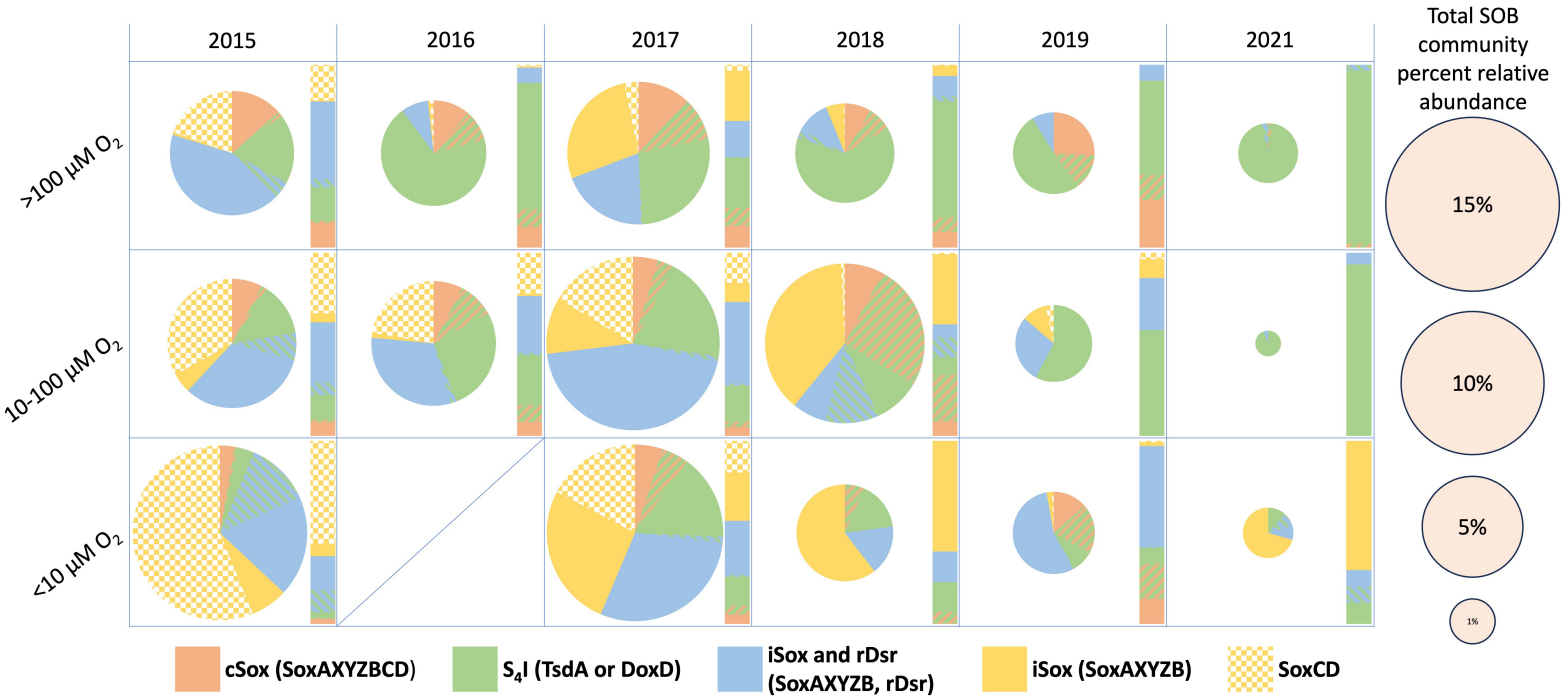
Pie charts (size proportional to SOB abundance of entire microbial community) and bar graphs (normalized to 100% of SOB community) showing the relative abundance of sulfur oxidizing pathways based on 16S rRNA abundance data from BML P1 samples in July and August of 2015–2019 and 2021. Samples were averaged according to year and categorized by dissolved oxygen concentrations.

From 2015 to 2021 the total relative abundance of SOB in BML reached as high as 29% and averaged 6.7% (Supplementary Figure 1). Therefore, while there was consistent presence of SOB, the overall relative abundance was smaller than mining environments where SOB have previously been studied (Liu, F. Y. L. et al., 2025; Liu et al., 2025; Twible et al., 2024; Whaley-Martin et al., 2023). The presence of SOB with varying metabolic pathways during summer stratification periods (July and August), varied year to year and with oxygen concentration. SOB possessing the first step of the S4I pathway (*tsdA* or *doxD*; Figures 2, 3) were present every year and were generally the most abundant genera occurring in more oxygenated (DO > 100 μM) surface waters (Figure 3). *Thiobacillus* spp. which have been reported to possess the iSox and rDSR pathway, were more sporadic in abundance but were still observed across years and a range of oxygen concentrations. *Thiobacillus* spp. were also the only SOB examined in the study that have been reported to be capable of catalyzing the second step of the S4I pathway (*tetH;* Reaction (e), Supplementary Table 1). Here, cSox genera (*soxAXYZBCD*; Figures 2, 3) were present at most oxygen concentrations in the BML water cap until 2021 when they were only present at very low abundance above 100 μM oxygen. iSox and rDSR genera (*soxAXYZB, dsrAB*; Figures 2, 3) were most abundant during the transition year and decreased in abundance during the post alum years. They were most abundant in the lower oxygen zones (DO < 100 μM) and tended to decrease in abundance as oxygen concentration increased (Figure 3). While *Sulfurimonas* spp. was one of the most abundant SOB pre- alum addition (2015–2016) and during the transition period (2017) in the lower oxygen waters (DO < 100 μM), its abundance decreased post-alum. This suggests that in addition to varying with oxygen concentrations, SOB communities were further influenced by biogeochemical changes associated with the alum addition that resulted in post-alum summer hypolimnetic anoxia.

As shown in Supplementary Figure 2, typically one SOB genus was the most abundant member for each pathway over the sampling period. *Algoriphagus* spp.*, Sulfuritalea* spp., and *Methylovulum* spp. were consistently the most abundant members of the S4I, rDSR, and iSox pathways respectively, while both *Hydrogenophaga* spp. and *Polynucleobacter* spp. were the dominant members of the cSox pathway (Supplementary Figure 2). Interestingly, while *Hydrogenophaga* spp. were prominent cSox SOB in all zones, *Polynucleobacter* spp. were only abundant in the higher oxygen zones (>10 μM DO) of BML after 2015. Similarly, *Hydrogenophaga* spp. were a prominent member of the S4I pathway however, only in the low oxygen zones (< 10 μM DO) in BML, with the exception of 2017.

The two genera identified as capable of only autotrophic sulfur oxidation metabolism, *Thiovirga* and *Halothiobacillus*, were generally not dominant members of their respective pathways with *Thiovirga* never comprising more than 25% of cSox SOB abundance, and *Halothiobacillus* only comprising greater than 25% of the cSox and S4I pathways in the 10–100 μM O_2_ zones in July and August of 2016 and 2018. There does not seem to be any discernable pattern in the changing abundance of these strictly autotrophic SOB however their overall abundance decreased over time, and both were absent or present at less than 0.1% of the overall community in July and August of 2019 and 2021. This suggests that post alum, the high carbon conditions of BML may be selecting for SOB capable of heterotrophy rather than strictly autotrophic SOB.

**Figure 4:**
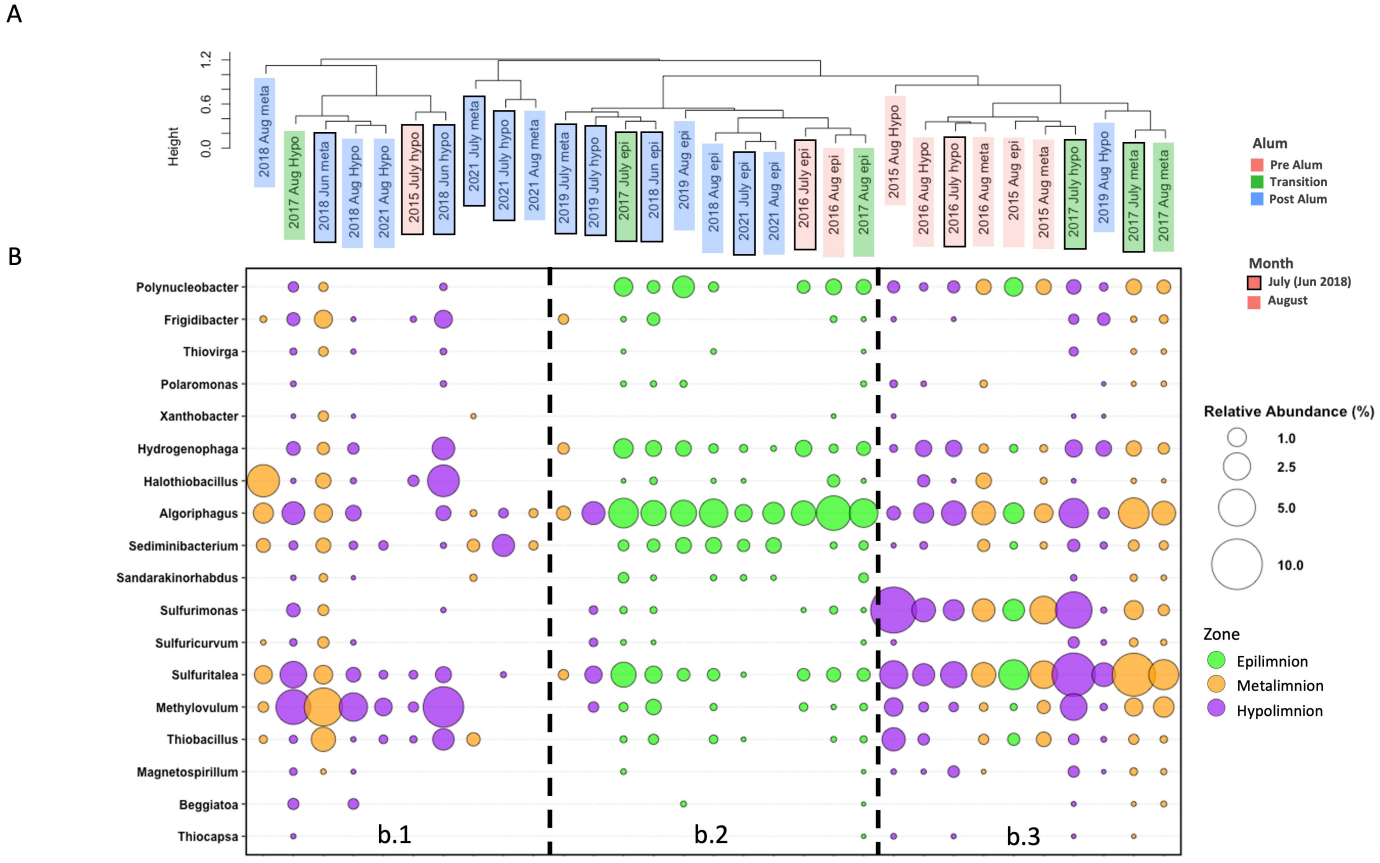
**(A)** UPGMA clustering of the average percent relative abundance of the SOB community for each thermal zone in July and August of 2015–2019 and 2021 at P1. **(B)** Bubble plot for the average percent relative abundance of the SOB community for each thermal zone in July and August of 2015–2019 and 2021 at P1.

Consistent with the notion that biogeochemical changes occurred in BML associated with the alum addition in September 2016, three SOB community clusters were identified using UPGMA hierarchical clustering on average SOB community abundance determined for P1 epilimnetic, metalimnetic, and hypolimnetic regions during July and August for 2015–2019, and 2021 (Figure 4A). Cluster b.1 consisted entirely of post-alum addition metalimnetic and hypolimnetic SOB communities (exception, July 2015 hypolimnion). The b.1 cluster was characterized by a higher abundance of *Methylovulum* spp. with inconsistent presence of other SOB (Figure 4B). Cluster b.2 consisted of all the epilimnetic communities over the sampling period, as well as the July 2019 metalimnetic and hypolimnetic communities. This cluster was characterized by a high abundance of *Algoriphagus* spp. and diminished abundance of other common SOB. Finally, cluster b.3 was comprised of pre-alum metalimnetic and hypolimnetic communities with two exceptions: the August 2015 epilimnetic community and the August 2019 hypolimnetic community. This cluster was characterized by high abundances of *Sulfuritalea* spp. and *Sulfurimonas* spp. with consistent presence of other SOB. These clusters indicate shifts in the BML SOB community occurred surrounding the alum addition. The clear distinction of the SOB communities specifically in the pre-alum and post-alum metalimnetic and hypolimnetic samples further indicates the largest impact occurred in the deeper waters (Figure 4) associated with the emergence of the hypolimnetic anoxic zone and altered biogeochemical cycling post-alum. This is reinforced by the single cluster of epilimnetic SOB communities across the entire pre—transition —post-alum time series. While the pre-alum epilimnetic samples are grouped together, they are monophyletic with the epilimnetic samples of other years, suggesting the alum had a smaller impact on the SOB community composition in the epilimnion where oxygen was consistently present at the highest observed concentrations.

### 3.3 Physicochemical conditions impacting SOB abundance and function

**Figure 5:**
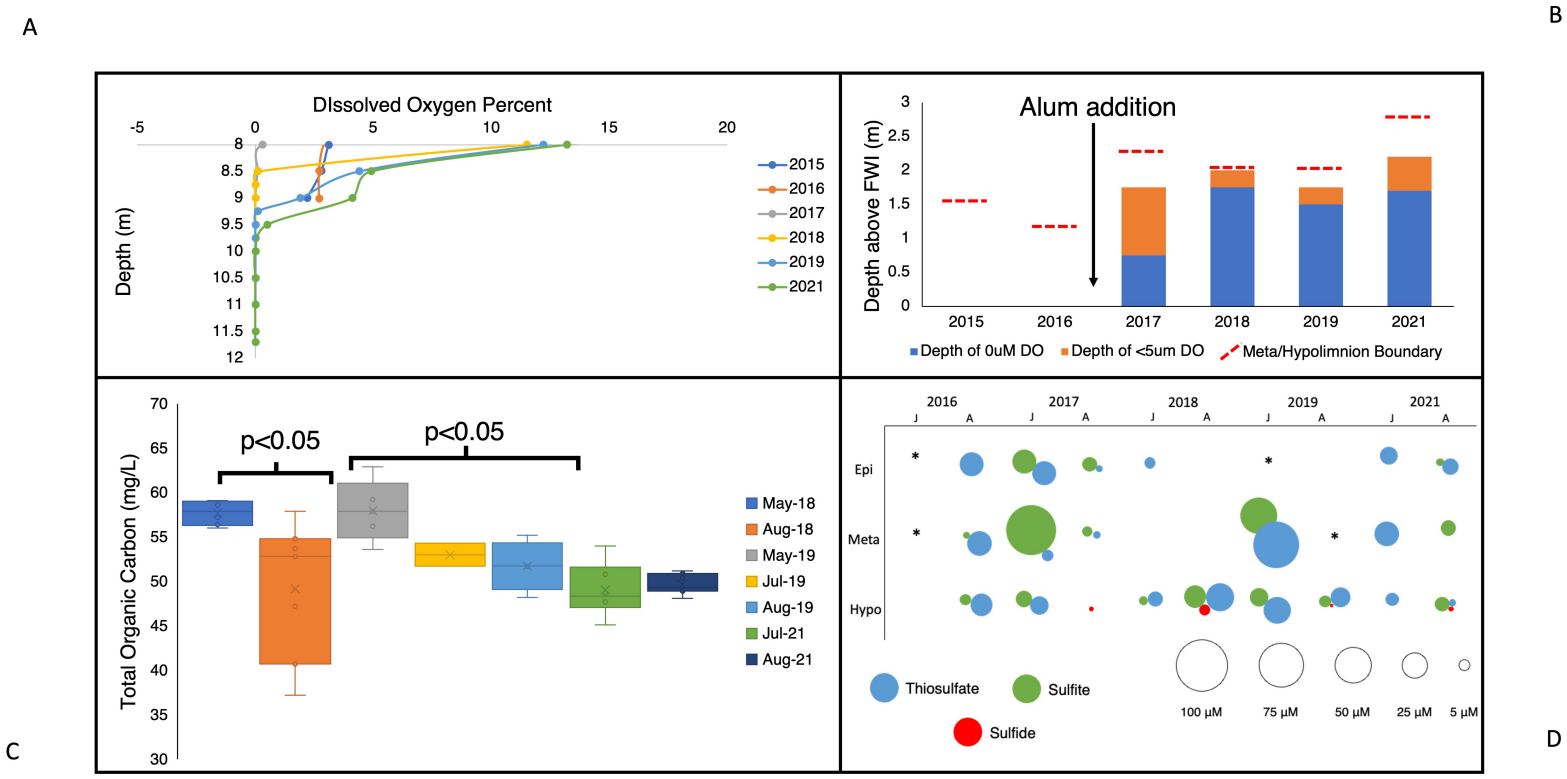
**(A)** Dissolved oxygen percent (%) in the BML hypolimnetic waters (8m–FWI) for August across study years (2015–2019, 2021). **(B)** Depth of anoxic zone (0 μM DO) and zone of <5 μM DO in comparison to the boundary of the metalimnion and hypolimnion. **(C)** Total organic carbon (TOC, mg/L) throughout the water column at P1 during spring (May) and summer months (July and August) in 2018, 2019, and 2021. **(D)** July and August epilimnetic, metalimnetic, and hypolimnetic mean sulfur species concentrations (sulfite, thiosulfate, sulfide) from 2016–2019 and 2021. ^*^SOI data unavailable for the specified month/thermal zone.

In addition to the metabolic repertoire of SOB and trends in their abundance, physicochemical and geochemical characteristics in BML also indicated ephemeral conditions conducive to active sulfur oxidation occurring in the water cap at certain times. While BML is not limited by total carbon, based on observed concentrations, much of the existing carbon is recalcitrant and not easily degraded by biological oxidation (Siddique and Kuznetsova, 2020) indicating high labile carbon substrates may be limiting in this system. Immediately following the alum addition, increased photic zone depth led to more primary production and the possibility of seasonal variation in labile organic carbon associated with algal growth, thereby impacting conditions for heterotrophic and autotrophic growth (Jessen et al., 2022). During the transition from spring turnover to summer stratification, settling of FFT particles mobilized throughout the water cap due to mixing (Yan et al., 2022) increased light penetration leading to greater primary production and higher concentrations of labile organic carbon. As summer progressed, this autochthonous biomass would facilitate the growth of heterotrophs, increase consumption of carbon, and lead to lower labile carbon conditions by late summer. This is evidenced by the significant decrease in TOC seen from May to August of 2018 and 2019 (*p* < 0.05, Figure 5C). This trend is likely reinforced by the resuspension of particulate organic matter during the spring turnover event, followed by their gradual settling. The lower labile carbon availability of late summer would reduce the competitive advantage of heterotrophic growth, and allow for more autotrophic growth in the water cap. This seasonal change in labile carbon content would favor SOB capable of heterotrophic growth during periods of high carbon, that could switch to autotrophic sulfur oxidation when organic carbon becomes limiting. Research has identified in some organisms the *sox* operon is induced via the presence of thiosulfate (Pyne et al., 2018). Therefore, there is the potential for thiosulfate to be oxidized regardless of labile organic carbon concentration and subsequently become the primary energy source when labile organic carbon is limiting.

Prior to the alum addition (2015–2016), oxygen was persistent to the FWI, as concentrations of oxygen consuming constituents (OCC, e.g., sulfide, methane, ammonia) mobilizing from the FFT into the water cap were not sufficient to result in anoxia. Previous studies have shown the stimulation of autochthonously produced biomass, post-alum, increased oxygen consumption associated with its subsequent decomposition that tipped the BML system into late summer hypolimnetic anoxia, observed for the first time in 2017 (alum addition in September 2016; Jessen et al., 2022; Yan et al., 2022). This anoxic zone grew larger in 2018 and recurred in the late summer of each year in this study (Figure 5A, B). A lack of oxygen in the bottom waters would restrict the depth at which aerobic heterotrophs could grow, favoring bacteria that have the capability to use other electron acceptors such as nitrate in the lowest waters. Another effect of this anoxic zone was expansion of SRB into the water cap and the subsequent detection of SRB-generated sulfide in the anoxic zone every year since 2017 (Figure 5D; Jessen et al., 2022). Additional sulfur species have also been regularly detected throughout the water cap including thiosulfate (Yan et al., 2022), a common substrate for the primary sulfur oxidation pathways, i.e., cSox, iSox, rDSR, and S_4_I (Figure 5D).

The use of alternative electron acceptors by SOB genera found in BML has also been observed previously. Nitrate use as an electron acceptor for thiosulfate oxidation has been observed in incubations inoculated with BML tailings directly demonstrating the anaerobic sulfur oxidation capabilities of BML SOB (Stasik et al., 2021). In other systems, SOB genera such as *Sulfuricurvum, Sulfurimonas, Thiobacillus*, and *Sulfuritalea* have also been observed to use nitrate to oxidize sulfur species including thiosulfate, elemental sulfur, and sulfide (Aminuddin and Nicholas, 1973; Biderre-Petit et al., 2011; Kojima and Fukui, 2011). The use of several other electron acceptors such as nitrite, ferric iron, and arsenate by the SOB genera observed in BML has also been documented (Aminuddin and Nicholas, 1973; Brock and Gustafson, 1976; Watanabe et al., 2017). These studies collectively suggest there is widespread potential for the anaerobic oxidation of sulfur using alternative electron acceptors under late summer BML water cap biogeochemical conditions of anoxia, lower carbon substrate availability, and micromolar concentrations of a variety of possible sulfur substrates.

**Figure 6:**
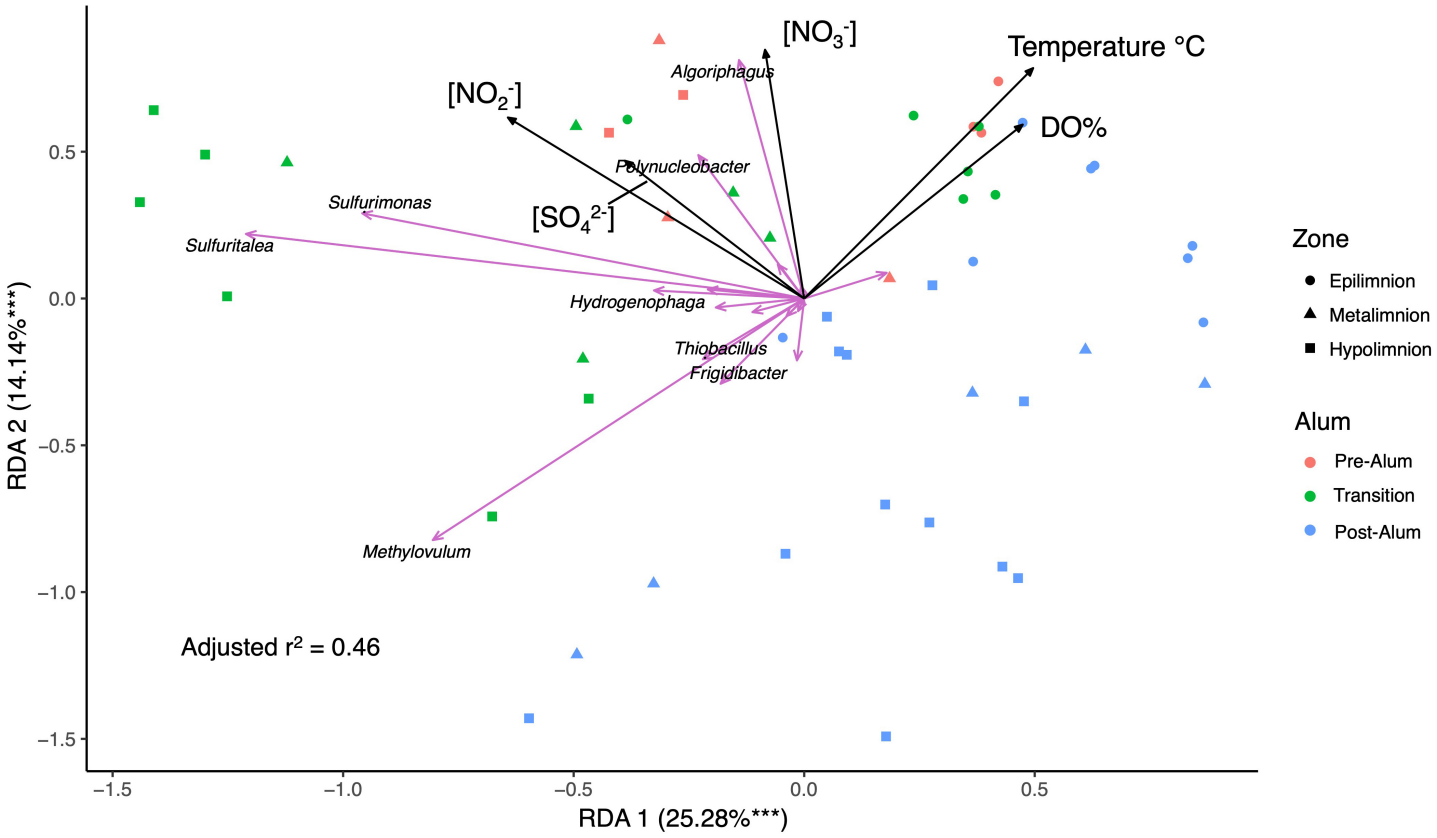
Redundancy Analysis (RDA) of BML P1 SOB community relative abundance, physicochemical data (temperature [°C], dissolved oxygen percent [DO%]), and geochemical data ([${\text{NO}}_{2}^{-}$], [${\text{NO}}_{3}^{-}$], and [${\text{SO}}_{4}^{\text{2}-}$]) in July and August of 2016–2019 and 2021 with the top 8 most significant SOB genera labeled. ^***^ *p* < 0.001.

Redundancy analysis (RDA) and stepwise selection of 11 environmental variables impacting the SOB community revealed DO, temperature, [${\text{NO}}_{2}^{-}$], [${\text{NO}}_{3}^{-}$] and [${\text{SO}}_{4}^{\text{2}-}$] were significant variables explaining SOB community variation with 39.4% of the variance explained in the first two axes (Figure 6). Interestingly, no specific sulfur-based electron donors were determined to be significant in explaining SOB variation and further investigation revealed no trend between SOB abundance and [${\text{SO}}_{3}^{\text{2}-}$], [S_2_O${\text{SO}}_{3}^{\text{2}-}$], or [ΣH_2_S]. This finding is in contrast to previous studies of metal mine tailings impoundments (TIs) which have found sulfur species such as thiosulfate to be a major factor impacting the endemic, autotrophic SOB communities (Liu et al., 2025; Twible et al., 2024). All epilimnetic samples clustered together, divergent from the metalimnetic and hypolimnetic samples that are separated into pre-alum and post-alum clusters, providing insights into the physicochemical and geochemical factors influencing BML SOB communities (Figure 6). The divergence of sample clusters is mostly driven by the presence of e^−^ acceptors, [${\text{NO}}_{2}^{-}$], [${\text{NO}}_{3}^{-}$] and DO, reflecting the shift of e^−^ acceptors during summer bottom water anoxia post alum (Figure 6).

[${\text{SO}}_{4}^{\text{2}-}$] varied over years in BML due to both operational changes and biogeochemical cycling. The concentration of sulfate in July and August of 2016 was 1.58 ± 0.18 mM, before increasing significantly in 2017 to 1.89 ± 0.10 mM (*p* < 0.001). [${\text{SO}}_{4}^{\text{2}-}$] then decreased to pre-alum levels in 2018 (1.53 ± 0.07 mM) and remained relatively stable until 2021 when the concentration decreased to 1.43 ± 0.03 mM. Elevated sulfate concentrations in 2017 were likely due to the September 2016 addition of a large quantity of aluminum sulfate (*X*Al(SO_4_)_2_ 12H_2_O, where *X* is a monovalent cation) to the BML water cap. While SRB have been detected in the water cap since 2017, the average (Sreact) in 2021 was the highest since 2016, suggesting that a combination of increasing SRB activity and/or decreasing SOB activity led to the accumulation of reactive sulfur (Jessen et al., 2022; Yan et al., 2024; Supplementary Figure 3). Nitrate and nitrite concentrations were also affected by the alum addition. Prior to the alum addition nitrate concentrations were relatively consistent across summer thermal zones, averaging a concentration of 29.4 ± 17.5 μM (Table Supplementary 2), which was followed by a mean 2017 summer water cap nitrate concentration of 41.9 ± 22.2 μM (Supplementary Table 2) during the transition year. In July and August of 2018 nitrate concentrations were below the limit of detection throughout the entire water cap, whereas in 2019 and 2021 the concentration of nitrate was stable through depth (54.9 ± 5.3 μM, average of epilimnion and metalimnion; Supplementary Table 2) until the hypolimnion at which point the concentration fell to below the limit of detection. The depth dependent trends of nitrate and oxygen concentrations observed in post-alum years (2018, 2019, and 2021) were consistent with its use as an electron acceptor in anoxic or microoxic zones. Nitrate would be used by anaerobic heterotrophs which has been widely reported (Wang et al., 2014; Xi et al., 2022) as well as by anaerobic SOB for sulfur oxidation, when suitable carbon sources become limiting to heterotrophy. The highest average summer water cap concentration of nitrite (5.0 ± 2.7 μM; Supplementary Table 2) occurred in 2016, and subsequently decreased each year, falling below detection from 2019 onwards. The decrease in nitrite concentrations in years post-alum corresponds with the observed gradual decrease in SOB abundance (Figure 3A). This suggests that nitrite may be an important electron acceptor for SOB, as has been observed in other SOB communities (Oberoi et al., 2021; Wang et al., 2021), without which both SOB abundance and activity decreased.

### 3.4 Diversity of BML SOB and the whole microbial community

**Figure 7:**
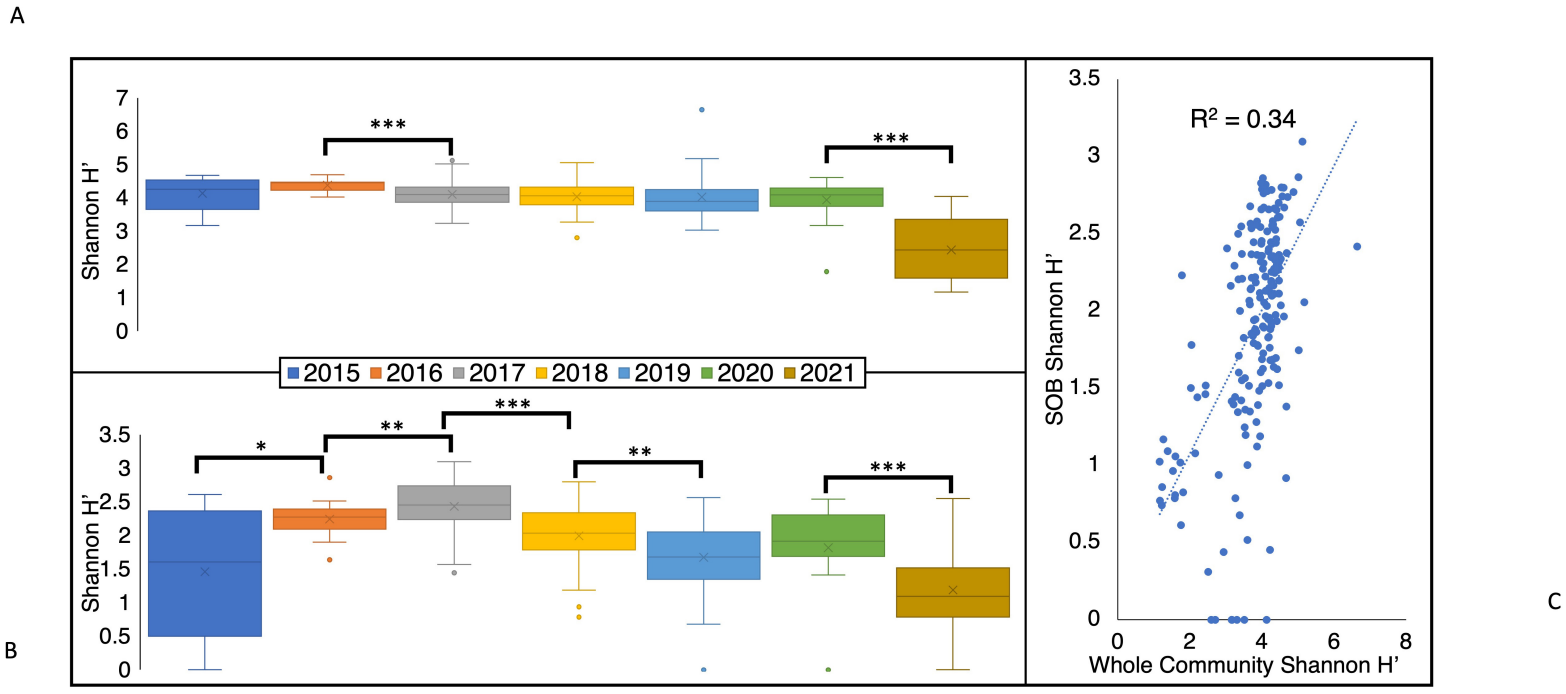
Box and whisker plots comparing the 2015–2021 annual **(A)** Shannon diversity index (H′) values for the total BML microbial community and **(B)** Shannon diversity index (H′) of the BML SOB community. **(C)** Scatter plot of Shannon diversity index (H′) values for the whole microbial community compared to the SOB community. *P*-values determined by Welch's *t*-test, and significance indicated by *p < 0.1, **p < 0.05, and ***p < 0.01. Box limits indicate the first and third quartile of each data set, with a line indicating the median and an “x” denoting the mean.

The average Shannon diversity index (H′) values of the total BML community (1.2 to 6.7) significantly decreased between 2016 (4.4 ± 0.2) and 2017 (4.1 ± 0.4, *p* < 0.01) immediately after the alum addition (September 2016; Figure 7A) and decreased again between the two latest years in this study, 2020 (3.9 ± 0.6) and 2021 (2.4 ± 0.9, *p* < 0.01) to a minimum that was significantly different from every other year (*p* < 0.01). In contrast to the total microbial community, the Shannon H′ diversity of the SOB community showed greater annual variability, significantly increasing from 2015 to 2017, and then decreasing in the following years (Figure 7B). Consistent with differential environmental influences shaping the SOB community relative to the whole BML community, only a minor relationship occurred between the whole community Shannon diversity and SOB community Shannon diversity indices (R^2^ = 0.34; Figure 7C). When compared to various literature samples, the Shannon diversity of the whole community for BML fell in the range of a previously studied flooded petroleum reservoir (H′ = 1.6–4.9, Supplementary Figure 4). BML samples that had the highest diversity were similar to values observed for Lake Shihou (H′ = 3.9–5.0), however it is more common for lakes to have higher diversity values (H′ = 5.5–8.9, Supplementary Figure 4) than those observed here. The lowest diversity values observed in BML (2021) was more similar to that reported for a base metal mine TI (H′ = 1.4–4.9, Supplementary Figure 4) indicating potential progression of decreasing BML bacterial diversity levels to resemble more niche environments. The highest Shannon diversity value in BML was 6.7 (Figure 7A) and was recorded in July 2019 at SP, which is a shallow littoral sampling site (~1.4 m) in the southeast corner of the lake (Figure 1A). Samples taken in May of 2019 at E16, a slightly deeper littoral site (~3.1 m; Figure 1A), also showed higher diversity values (average H′ = 5.1) compared to pelagic samples taken in May of 2019 (average H′ = 4.3). Consistent with their littoral nature, these sites occurring in the epilimnetic and photic region of the lake were consistently lighted and oxygenated during the summer months, resulting in higher productivity and bacterial cycling of organic carbon and nutrients.

### 3.5 Changes in prevalence of microbial sulfur oxidation pathways

**Figure 8:**
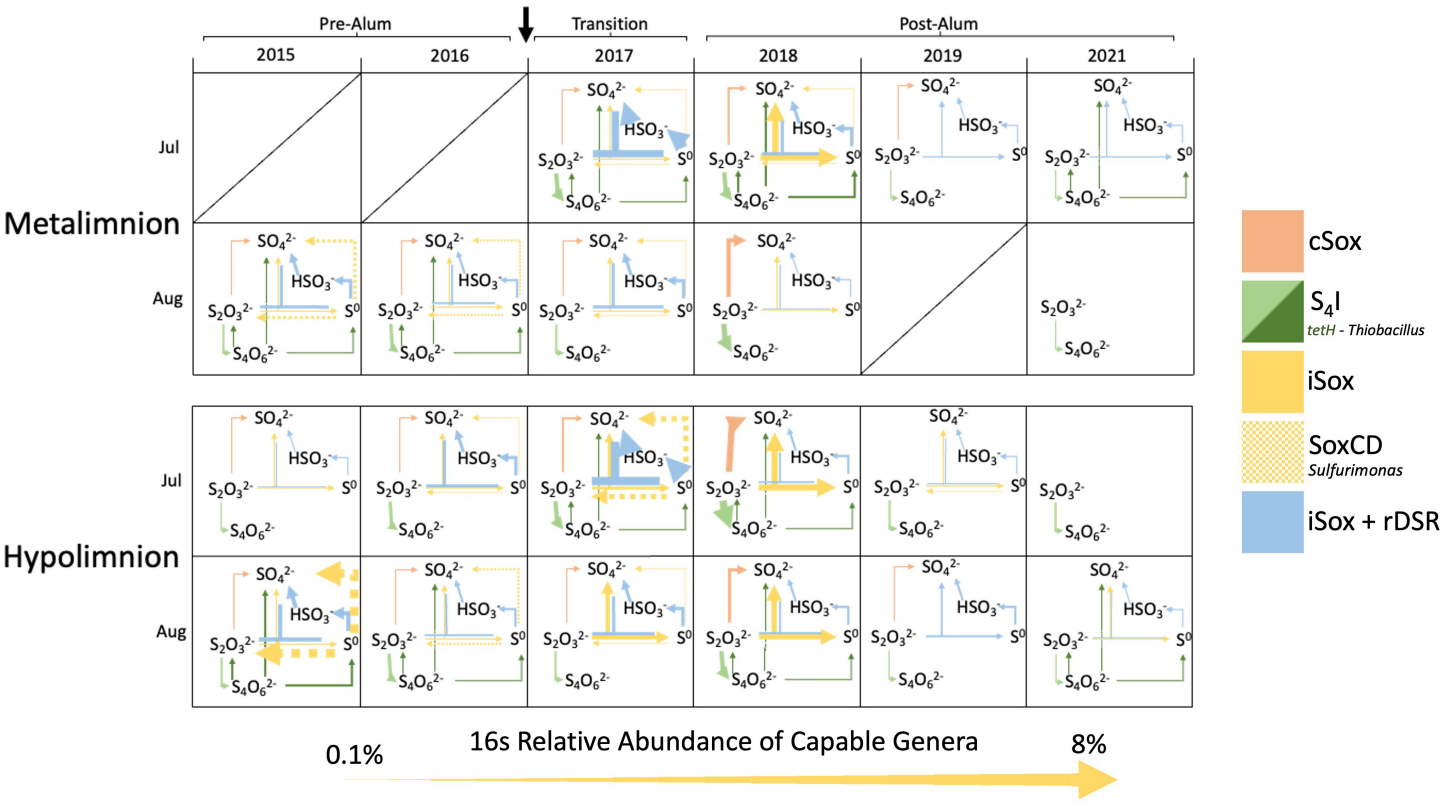
Sulfur oxidizing pathways occurring (>0.1%) in BML in the P1 metalimnion and hypolimnion for July and August for 2015–2019, and 2021. Estimated abundance of the pathway (>0.1%) corresponds with the thickness of the arrow and was calculated based on the sum of the average 16S rRNA abundance of SOB genera that possessed each pathway based on metagenomic data and existing literature (Figure 2).

In 2017 and 2018, meta and hypolimnetic SOB abundance was higher in July than in August, with comparable abundances in the epilimnion of both months (Figure 8; Supplementary Figure 5). The highest abundance of genera possessing the cSox pathway was in the hypolimnion of July 2018, followed by the metalimnion of August 2018. As the majority of the hypolimnion became anoxic in August of 2018, the migration of cSox up into the metalimnion suggests a reliance on oxygen to carry out their metabolism. During this time, *Halothiobacillus* was the most abundant cSox SOB, and the propensity for *Halothiobacillus* to oxidize thiosulfate under more oxic conditions has also been noted in base metal mine TIs (Whaley-Martin et al., 2023). While the decrease in oxygen impacted hypolimnion SOB communities, metalimnion SOB relative abundance also decreased between July and August despite the constant presence of oxygen. This decrease in abundance may be due to the decrease in labile organic carbon and the switch to autotrophic sulfur oxidation which typically generates less energy than heterotrophy. Additionally, the restricted pool of reduced sulfur substrates may also be a limiting factor for SOB growth in July and August, furthering the decrease in abundance when they are more reliant on autotrophic sulfur oxidation. In 2019 and 2021 there is low abundance of SOB in July and therefore a much smaller difference in abundance from July to August (Figure 8). Overall, the larger abundances of iSox, S_4_I, and rDSR SOB, compared to cSox SOB, allows for the generation and recycling of SOI in BML rather than more well-constrained complete oxidation of thiosulfate to sulfate. This likely aids in the persistence of SOB during periods of reduced labile organic carbon and preserves S substrate for both SOB and SRB growth.

### 3.6 Evidence of seasonal conditions causing ephemeral switches in sob from heterotrophy to autotrophic sulfur oxidation

**Figure 9:**
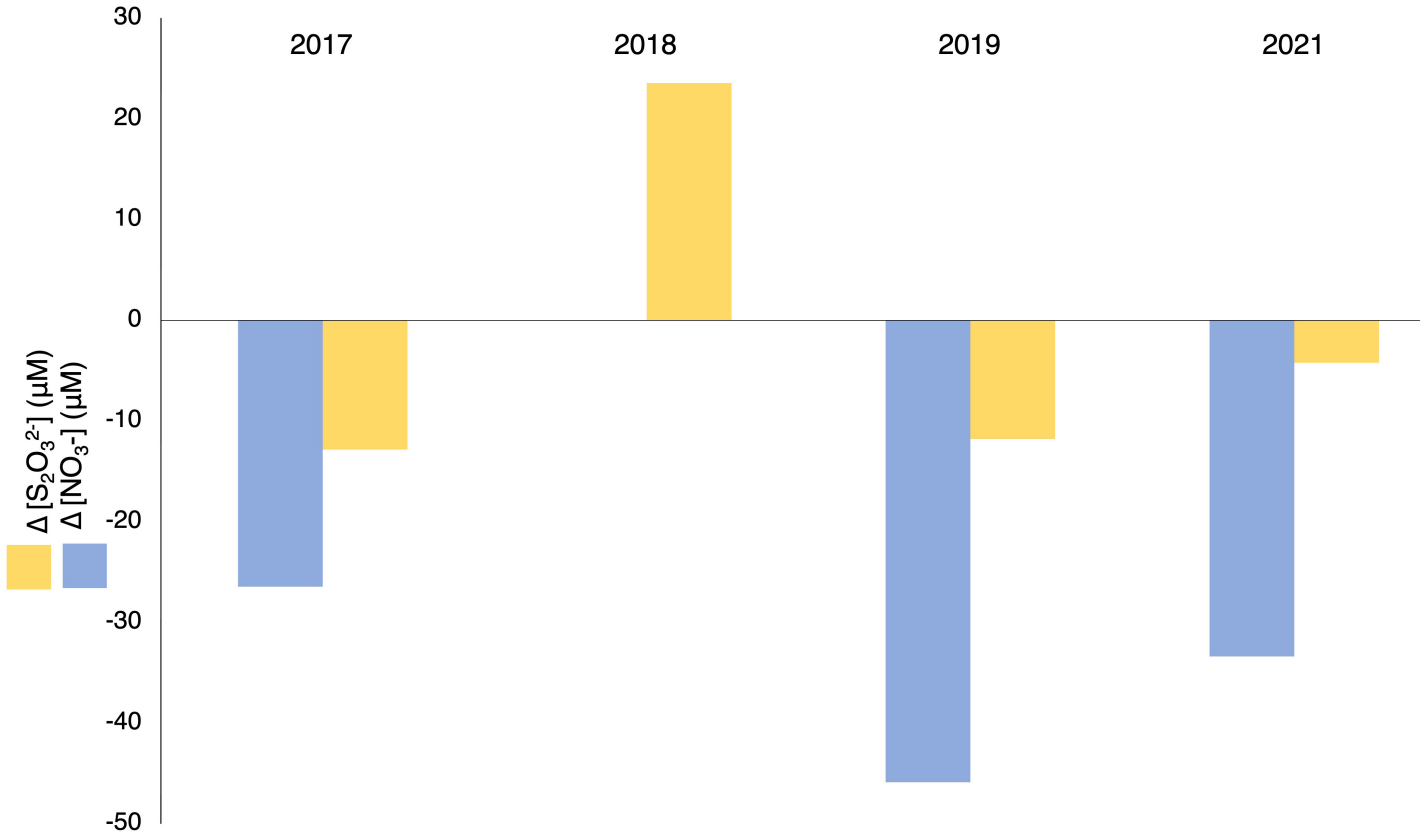
Mean delta observed in post-alum P1 hypolimnetic nitrate and thiosulfate concentrations between July and August (2017–2019, 2021). [${\text{NO}}_{3}^{-}$] in 2018 was below the limit of detection and precluded calculation of Δ[${\text{NO}}_{3}^{-}$] for that year.

Evidence of sulfur oxidation was observed in the hypolimnion of July and August in years post-alum addition. Both nitrate and thiosulfate concentrations decreased in the hypolimnion from July to August of every year, except for 2018, consistent with SOB thiosulfate oxidation coupled to nitrate reduction (Figure 9). In 2018 nitrate was undetectable in the water column, coincident with an increase in thiosulfate in the hypolimnion. This suggests thiosulfate was not being consumed by SRB and nitrate was key to the consumption of thiosulfate. The ratio of nitrate and thiosulfate losses between July and August were higher than those calculated for nitrate to nitrogen gas dependent oxidation of thiosulfate, indicating more nitrate was being consumed than could be accounted for by thiosulfate oxidation coupled to complete denitrification alone (Equation 1, Supplementary Figure 6).


(1) $$\begin{array}{l} 8NO_{3}^{-}+5S_{2}O_{3}^{2-}+H_{2}O⇌4N_{2}+10SO_{4}^{2-}+2H^{+} \end{array}$$

(2) $$\begin{array}{l} 4NO_{3}^{-}+\;S_{2}O_{3}^{2-}+\;H_{2}O\;⇌4NO_{2}^{-}+2SO_{4}^{2-}+2H^{+} \end{array}$$

While nitrate may be reduced further to ammonia rather than nitrogen gas, this would yield more electrons, lowering the theoretical ratios of nitrate and thiosulfate consumed even further from those observed in BML (Supplementary Figure 7). However, it is common for bacteria to lack the complete set of genes required for the reduction of nitrate to nitrogen gas, leading to the formation of several intermediate nitrogen species (Graf et al., 2014; Lycus et al., 2017; Roco et al., 2017; Zhang I. H. et al., 2023). Conversion of nitrate to nitrite, nitric oxide, and nitrous oxide would all yield higher ratios of nitrate consumed per thiosulfate, through the cSox pathway (Equation 2, Supplementary Figure 7). While observed ratios of nitrate to thiosulfate consumed in BML suggested incomplete denitrification, this ratio increased over time and exceeded all theoretical values in 2021. These observed comparative changes in the concentrations ratios of these two compounds suggest increasing heterotrophic competition for nitrate with SOB, potentially limiting the ability of SOB to use this metabolism and explaining their diminishing abundance over time. This may reflect more rapid anoxic zone establishment leading to simultaneous anoxia and labile organic carbon, whereas previously it was likely labile carbon was limiting in the anoxic zone allowing for increased growth of SOB.

In addition to data from BML, enrichments using BML water and sulfur oxidation media were observed as growing SOB that have been previously observed as being capable of heterotrophy, further demonstrating their ability to oxidize sulfur (Supplementary Figure 8). As there is the potential for simultaneous activity of several different sulfur oxidation pathways, we cannot identify the exact pathways being used at BML, however the results of previous studies suggest complete thiosulfate oxidation to sulfate is likely occurring (Stasik et al., 2021). In 2018 when anaerobic sulfur oxidation would have been greatly reduced associated with undetectable nitrate concentrations (Supplementary Table 2), the anoxic zone occupied the largest percent of the hypolimnion over the time series (Figure 5B). These results suggest anaerobic SOB potentially mitigate sulfur oxidation risks to BML oxygen levels through their pre-emptive removal of these reduced sulfur compounds in the lower suboxic-anoxic waters.

**Figure 10:**
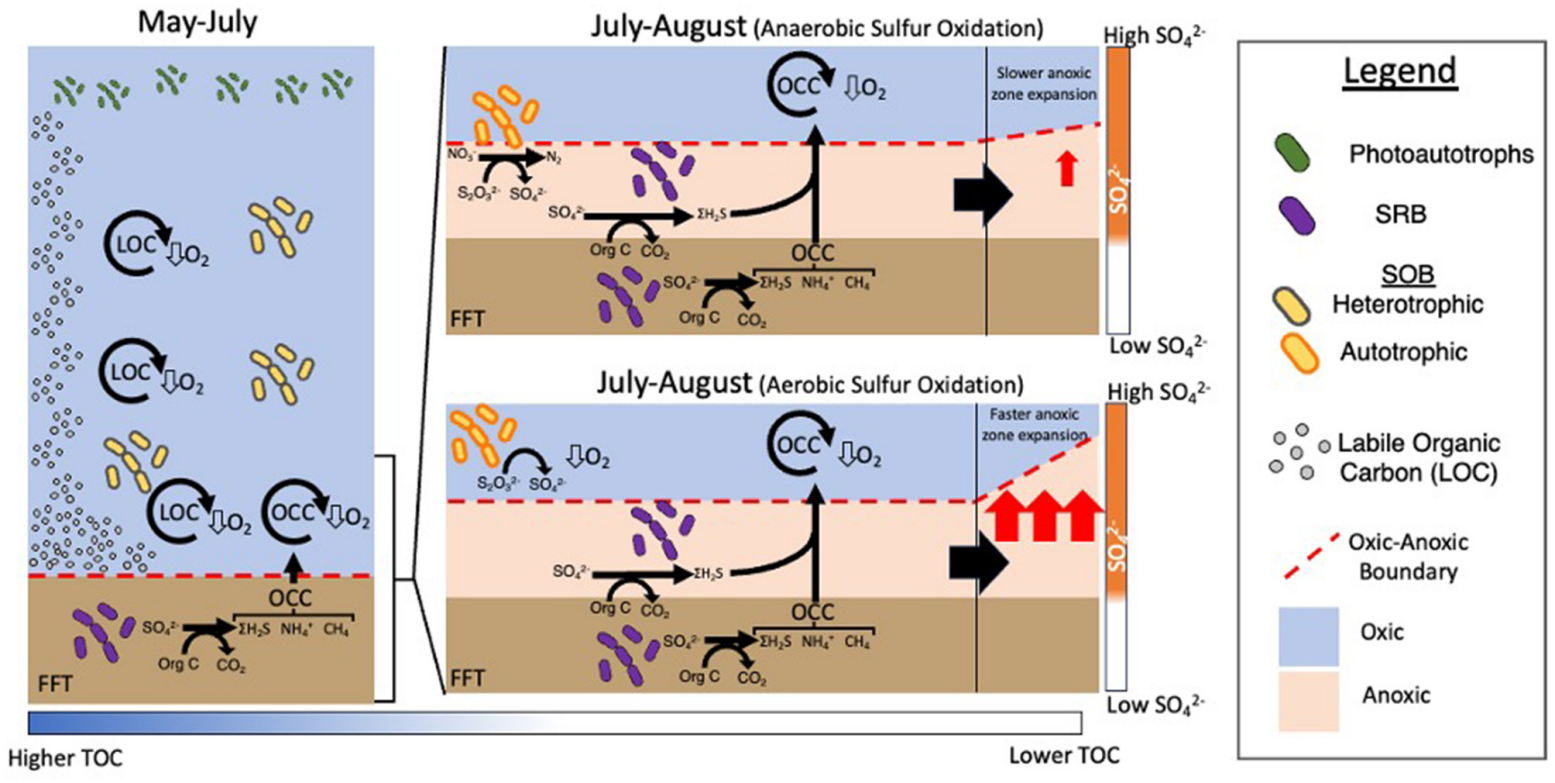
Schematic of the interaction between physico/geochemistry and the transition from predominantly heterotrophic to sulfur oxidation processes in BML being carried out by SOB in May compared to late summer (July and August).

Collectively these results reveal an interactive carbon-sulfur biogeochemical cascade was triggered by the alum addition which is dynamically reflected in SOB community changes and oxygen concentrations in BML. The alum addition increased water clarity, enabling greater algal biomass production, which in turn, supported greater aerobic heterotrophic degradation in the water column (Figure 10). SOB genera capable of heterotrophy were likely to be participating in heterotrophy while labile carbon was plentiful. As the summer progressed, the decomposition of labile organic matter along with cycling of OCC mobilizing from the FFT, consumed more oxygen than could be replenished from the upper waters (Arriaga et al., 2019), leading to the formation of an anoxic zone expanding from the FWI. This then allowed for the migration of SRB from the FFT into the water cap where sulfate was more plentiful, and sulfide was generated directly in the water cap. The on-going consumption of labile organic carbon led to lower concentrations, which when paired with increasing suboxic-anoxic conditions made the bottom waters unsuitable for aerobic heterotrophy. These conditions created a specific biogeochemical window in which it was advantageous for BML SOB to switch to anaerobic sulfur oxidation as evidenced by the simultaneous consumption of nitrate and thiosulfate in the July-August hypolimnetic waters post-alum addition. The anaerobic oxidation performed by SOB would then mitigate the oxygen consumption potential of reduced sulfur species generated in the water column. There is also the possibility of micro-aerophilic SOB occupying the micro-oxic waters just above the anoxic zone where autotrophy may still be favored. In this case, the added consumption of oxygen by SOB would further increase the expansion of the anoxic zone (Figure 10). Therefore, determining the electron acceptor use of SOB will be critical to managing oxygen risks in BML.

## 4 Conclusion

After an alum amendment in 2016, the bottom waters of BML became anoxic during peak summer stratification from 2017 to 2021 as a result of the physical and biogeochemical processes occurring in the lake. While the potential for SOB in BML to consume oxygen has been explored, the intricacies of the community has yet to be fully understood. In this study, we revealed a dynamic coalition of SOB communities with potential for heterotrophic or autotrophic activity depending on the oxygen and carbon conditions. Based on both literature and MAG data, SOB in BML were found to dynamically possess the genetic machinery associated with all three primary sulfur oxidation pathways (Sox, rDSR, S_4_I), all of which have different implications for oxygen consumption in BML. Specifically, *Algoriphagus* spp., *Sulfuritalea* spp., and *Methylovulum* spp., were found to the most abundant genera possessing the S_4_I, rDSR and iSox pathways respectively. While abundance of cSox was consistently low *Hydrogenophaga* spp., and *Polynucleobacter* spp., were the most dominant cSox SOB in BML. Previous literature identified the majority of BML SOB to be capable to heterotrophy, allowing for mixotrophic metabolism, where sulfur oxidation was used under certain conditions. Consumption of thiosulfate and nitrate suggests transient microbial anaerobic sulfur oxidation occurred specifically in the post-alum late summer hypolimnion driven by carbon limitation and anoxia that potentially mitigated the further expansion of the anoxic zone. Given the on-going changes observed in BML biogeochemical cycling, post commissioning in 2013, further work is needed to monitor the continued development of SOB in this system and understand their possible implications for oxygen consumption in this pilot pit lake that is being assessed as a reclamation strategy for FFT in AOSR.

## Data Availability

The 16S rRNA sequencing data generated from this study is available in the NCBI database under Bioproject PRJNA552483.
