# Supplementary material for: Sulfur oxidation and implications for oxygen consumption in Base Mine Lake, the first pilot oil sands pit lake in the Athabasca oil sands region
Source: Front Microbiol. 2025 Oct 21;16:1662147. doi: 10.3389/fmicb.2025.1662147 (PMC12584484; doi:10.3389/fmicb.2025.1662147)
Supplement: Supplementary file 1 [file Data_Sheet_1.docx]

Supplementary Material


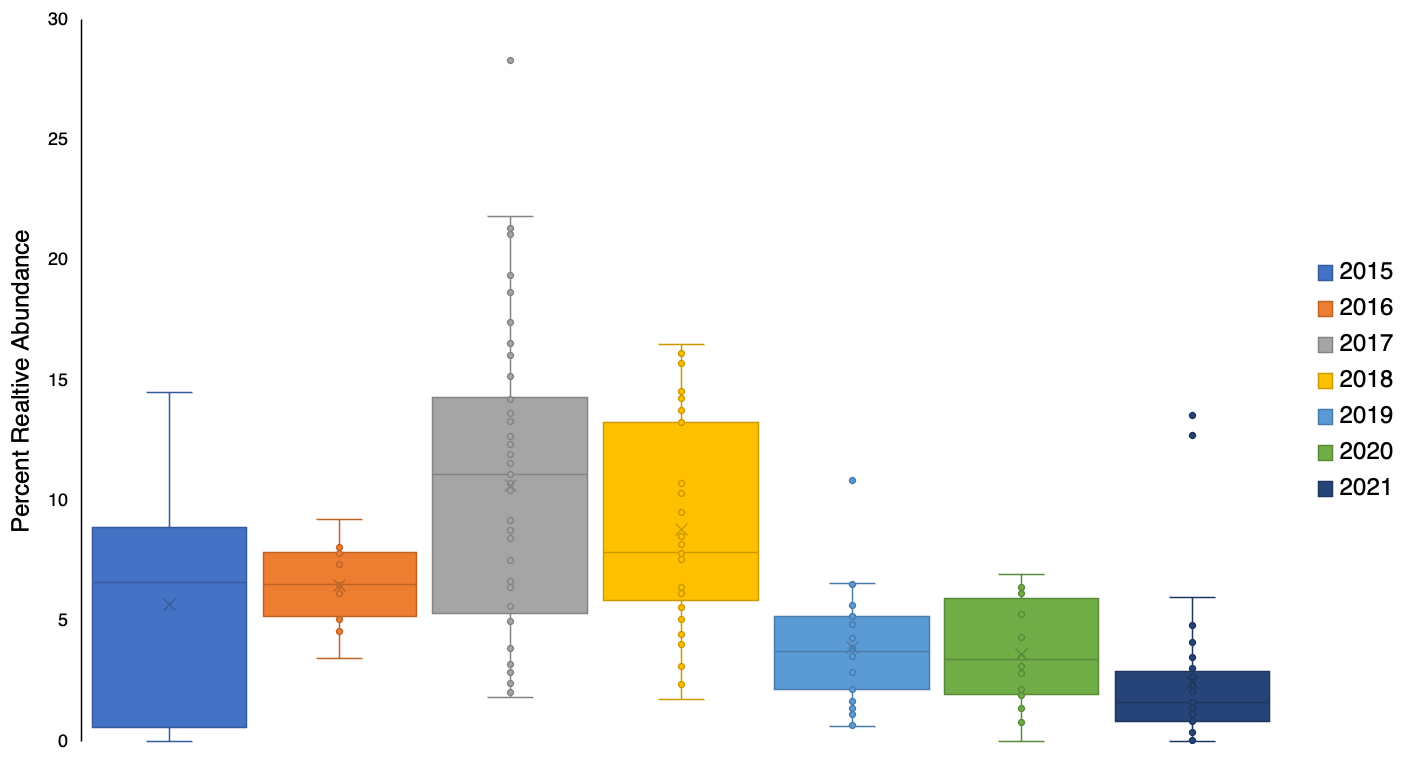


Figure S1. Percent relative abundance of SOB from 2015 to 2021. Box limits indicate the first and third quartile of each data set, with a line indicating the median and an “x” denoting the mean.


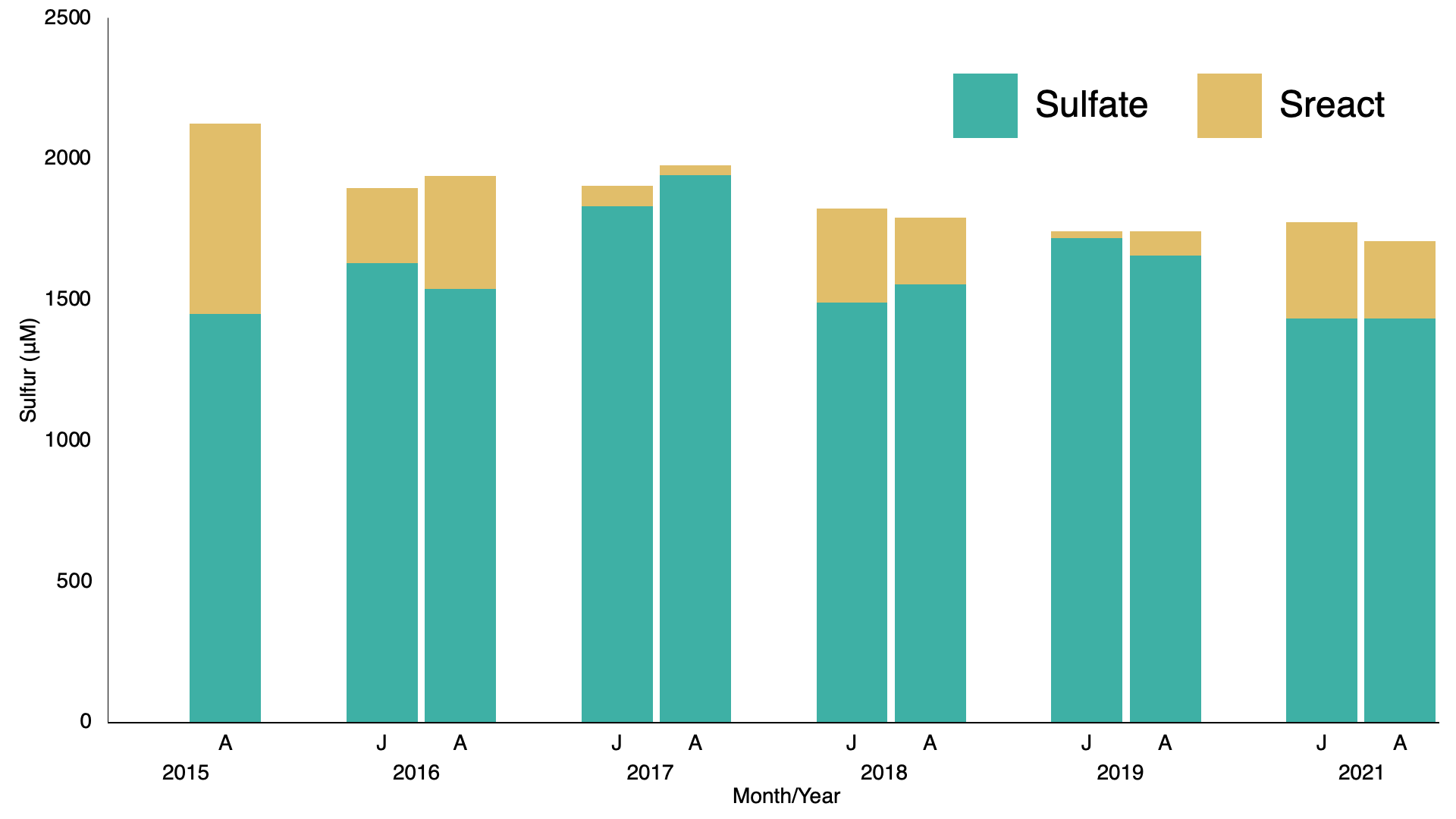


Figure S2. Average concentrations of Sreact and Sulfate in BML at P1 in July (J) and August (A) from 2015-2019 and 2020.


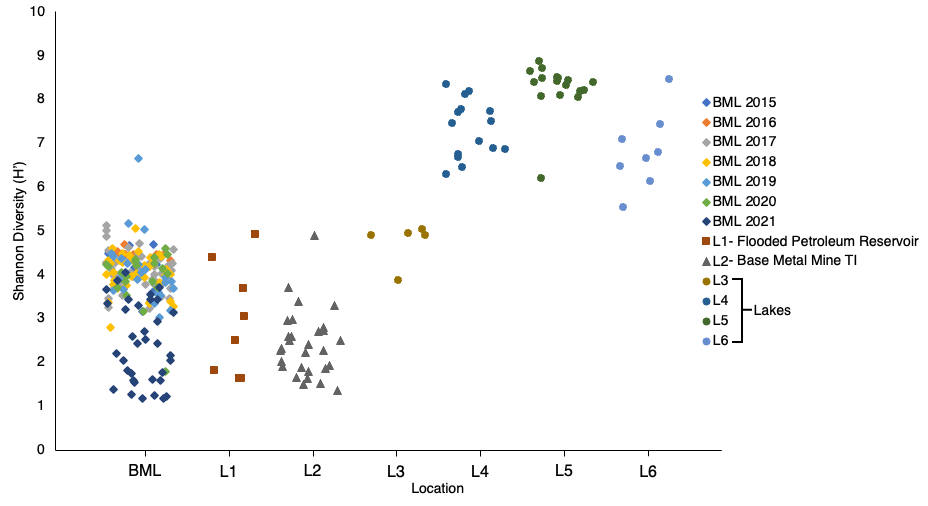


Figure S3. Shannon H' diversity of the BML bacterial community over time and other aquatic environments sorted according to location. L1 is a flooded petroleum reservoir (Tian et al., 2017), L2 is the TI (Tailings Impoundment) of a base metal mine (Whaley-Martin et al., 2023), and L3-L6 are freshwater lakes (Deng et al., 2021; Ji et al., 2019; Ozbayram et al., 2022; Shahraki et al., 2021).

Figure S4. Observed consumed nitrate to thiosulfate ratios in the BML July- August hypolimnion compared to theoretical values based on balanced half reactions. Theoretical values were determined based on reduction of nitrate to nitrogen gas.

Figure S5. Observed consumed nitrate to thiosulfate ratios in the BML July- August hypolimnion compared to theoretical values based on balanced half reactions of varying levels of nitrate reduction resulting in nitrite, nitric oxide, nitrous oxide, nitrogen gas, and ammonia. Theoretical values were determined based on oxidation of thiosulfate to sulfate.


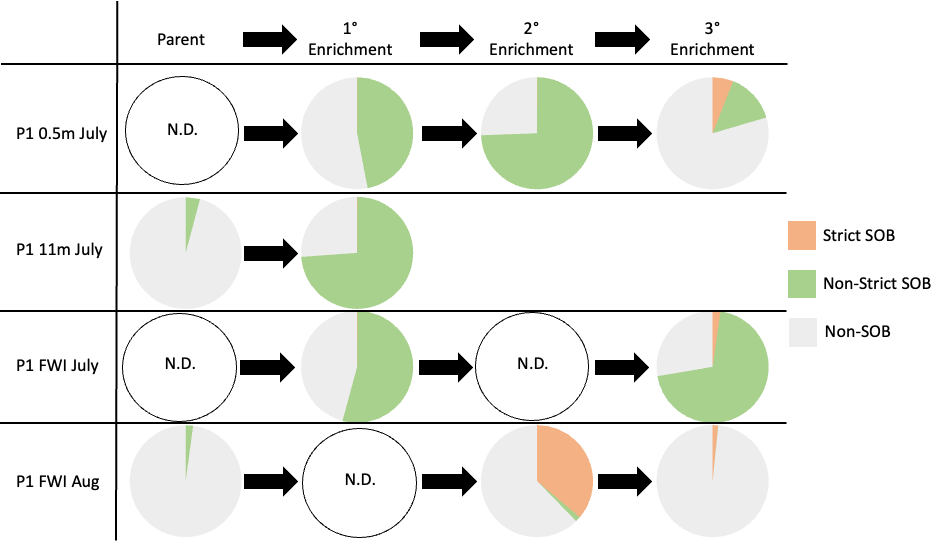


Figure S6. 16s rRNA data of parent and enrichment SOB communities. Enrichments were performed serially with enrichment 1 used as the inoculant for enrichment 2, etc. Samples for which no data was available are marked as N.D.. Non-strict SOB were defined as having an example of non-sulfur oxidation metabolism in the literature.

**Table S1**. Sulfur oxidation pathways, genes, reactions examined in this study.

| Pathway | Gene(s) | Reaction |  |
| --- | --- | --- | --- |
| cSox | *soxAXYZBCD* | $S_{2}O_{3}^{2-} 2SO_{4}^{2-}$ | (a) |
| iSox | *soxAXYZB* | $S_{2}O_{3}^{2-} SO_{4}^{2-}+S^{0}$ | (b) |
| rDsr | *dsrAB* | $S^{0} SO_{4}^{2-}$ | (c) |
| S4I (step 1) | *tsdA* or *doxD* | $S_{2}O_{3}^{2-} S_{4}O_{6}^{2-}$ | (d) |
| S4I (step2) | *tetH* | $S_{4}O_{6}^{2-} SO_{4}^{2-}+ S_{2}O_{3}^{2-}+ S^{0}$ | (e) |

Table S2. BML nitrate concentrations at P1 during July and August from 2015-2019 and 2021.

| Year | Month | Day | Depth | Nitrate (μM) | |
| --- | --- | --- | --- | --- | --- |
|  |  |  |  | Mean | Std Dev |
| 2015 | 8 | 18 | 1.5 | 24.2 | 0 |
| 2015 | 8 | 18 | 6.5 | 16.1 | 0 |
| 2015 | 8 | 18 | 8.5 | 16.1 | 0 |
| 2016 | 7 | 6 | 1.5 | 85.2 | 3.9 |
| 2016 | 7 | 7 | 8 | 23.5 | 2.1 |
| 2016 | 7 | 7 | 8.5 | 19.7 | 0 |
| 2016 | 7 | 7 | 9 | 20.1 | 1.4 |
| 2016 | 7 | 7 | 9.25 | 32.8 | 5.7 |
| 2016 | 8 | 4 | 0 | 16.7 | 13.9 |
| 2016 | 8 | 4 | 1.5 | 28.1 | 4.7 |
| 2016 | 8 | 4 | 4.5 | 22.3 | 1.8 |
| 2016 | 8 | 4 | 6 | 22.9 | 7.9 |
| 2016 | 8 | 5 | 6.5 | 45.7 | 11.1 |
| 2016 | 8 | 5 | 7.5 | 37 | 4.5 |
| 2016 | 8 | 5 | 9.3 | 30.4 | 23.3 |
| 2017 | 7 | 11 | 0 | 65.4 | 9.6 |
| 2017 | 7 | 11 | 1.5 | 61.4 | 3.2 |
| 2017 | 7 | 10 | 4.5 | 65.4 | 0.9 |
| 2017 | 7 | 12 | 5.5 | 73.5 | 6.6 |
| 2017 | 7 | 11 | 6.5 | 63.4 | 6 |
| 2017 | 7 | 12 | 7.5 | 27.3 | 5 |
| 2017 | 7 | 11 | 8 | 43.1 | 14.5 |
| 2017 | 7 | 12 | 8.5 | 36.4 | 1 |
| 2017 | 7 | 12 | 9 | 25.9 | 9 |
| 2017 | 7 | 12 | 9.5 | 29.8 | 8.8 |
| 2017 | 8 | 2 | 0 | 48.9 | 2.6 |
| 2017 | 8 | 2 | 1.5 | 54.5 | 2.7 |
| 2017 | 8 | 2 | 4.5 | 55.1 | 1.2 |
| 2017 | 8 | 2 | 5.5 | 52.3 | 1.7 |
| 2017 | 8 | 2 | 6.5 | 35.8 | 3.1 |
| 2017 | 8 | 1 | 7.5 | 22.5 | 8.6 |
| 2017 | 8 | 1 | 7.75 | 21.3 | 1.2 |
| 2017 | 8 | 1 | 8 | 14.7 | 3.8 |
| 2017 | 8 | 1 | 9.75 | <LOD |  |
| 2018 | 6 | 26 | 4.5 | <LOD |  |
| 2018 | 6 | 26 | 7.5 | <LOD |  |
| 2018 | 6 | 27 | 8.5 | <LOD |  |
| 2018 | 6 | 28 | 9 | <LOD |  |
| 2018 | 6 | 27 | 10 | <LOD |  |
| 2018 | 7 | 31 | 0 | <LOD |  |
| 2018 | 7 | 31 | 1.5 | <LOD |  |
| 2018 | 7 | 31 | 4.5 | <LOD |  |
| 2018 | 7 | 31 | 7.5 | <LOD |  |
| 2018 | 8 | 1 | 8.75 | <LOD |  |
| 2018 | 8 | 1 | 9.75 | <LOD |  |
| 2018 | 8 | 1 | 10.5 | <LOD |  |
| 2019 | 7 | 9 | 7.5 | 63.2 | 0.2 |
| 2019 | 7 | 9 | 11 | 60.3 | 0.8 |
| 2019 | 7 | 29 | 0.5 | 66.7 | 1.8 |
| 2019 | 7 | 29 | 9 | 57.6 | 1.3 |
| 2019 | 7 | 29 | 9.5 | <LOD |  |
| 2019 | 7 | 29 | 10 | <LOD |  |
| 2019 | 7 | 29 | 11 | <LOD |  |
| 2021 | 7 | 6 | 0.5 | 51.6 | 2.3 |
| 2021 | 7 | 6 | 11 | 47.7 | 2.6 |
| 2021 | 7 | 20 | 0.5 | 55.5 | 1.9 |
| 2021 | 7 | 20 | 5 | 55.8 | 1.5 |
| 2021 | 7 | 20 | 7.5 | 53.5 | 2.1 |
| 2021 | 7 | 20 | 10 | 51 | 0.7 |
| 2021 | 8 | 5 | 0.5 | 53.7 | 0.9 |
| 2021 | 8 | 5 | 3.5 | 53.5 | 1 |
| 2021 | 8 | 5 | 5.5 | 53.9 | 1.6 |
| 2021 | 8 | 5 | 7.5 | 52.2 | 2.1 |
| 2021 | 8 | 5 | 9.5 | 47.8 | 2 |
| 2021 | 8 | 5 | 11.5 | <LOD |  |
| 2021 | 8 | 5 | 11.7 | <LOD |  |

**References:**

Deng, R., Chen, X., Qiu, L. P., Chen, J. Z., & Meng, S. L. (2021). Bacterial Community Structure and Diversity in the Aqueous Environment of Shihou Lake and its Relationship with Environmental Factors. *Indian Journal of Microbiology*, *61*(4), 475–486. https://doi.org/10.1007/S12088-021-00974-Y

Ji, B., Liang, J., Ma, Y., Zhu, L., & Liu, Y. (2019). Bacterial community and eutrophic index analysis of the East Lake. *Environmental Pollution*, *252*(Pt A), 682–688. https://doi.org/10.1016/J.ENVPOL.2019.05.138

Ozbayram, E. G., Köker, L., Çam, A. O., Akçaalan, R., & Albay, M. (2022). Temporal and Spatial Variations of the Bacterial Diversity in a Deep Alkaline Lake. *Water*, *14*(24), 4097. https://doi.org/10.3390/W14244097

Shahraki, A. H., Chaganti, S. R., & Heath, D. (2021). Spatio-temporal dynamics of bacterial communities in the shoreline of Laurentian great Lake Erie and Lake St. Clair’s large freshwater ecosystems. *BMC Microbiology*, *21*, 253. https://doi.org/10.1186/S12866-021-02306-Y

Tian, H., Gao, P., Chen, Z., Li, Y., Li, Y., Wang, Y., Zhou, J., Li, G., & Ma, T. (2017). Compositions and abundances of sulfate-reducing and sulfur-oxidizing microorganisms in water-flooded petroleum reservoirs with different temperatures in China. *Frontiers in Microbiology*, *8*, 143. https://doi.org/10.3389/FMICB.2017.00143

Whaley-Martin, K. J., Chen, L. X., Nelson, T. C., Gordon, J., Kantor, R., Twible, L. E., Marshall, S., McGarry, S., Rossi, L., Bessette, B., Baron, C., Apte, S., Banfield, J. F., & Warren, L. A. (2023). O2 partitioning of sulfur oxidizing bacteria drives acidity and thiosulfate distributions in mining waters. *Nature Communications*, *14*(1), 2006. https://doi.org/10.1038/s41467-023-37426-8
